# Integration of HIV, Hepatitis B, and C, and sexually transmitted infections services: A scoping review of the benefits and challenges

**DOI:** 10.1371/journal.pone.0348073

**Published:** 2026-05-07

**Authors:** Parya Jangipour Afshar, Vahid Yazdi-Feyzabadi, Zahra Abdolahinia, AliAkbar Haghdoost, Jaason M. Geerts, Reza Goudarzi, Katayoun Tayeri, Babak Eshrati, Hamid Sharifi

**Affiliations:** 1 HIV/STI Surveillance Research Center, and WHO Collaborating Center for HIV Surveillance, Institute for Futures Studies in Health, Kerman University of Medical Sciences, Kerman, Iran; 2 Health Services Management Research Center, Institute for Futures Studies in Health, Kerman University of Medical Sciences, Kerman, Iran; 3 Student Research Committee, Kerman University of Medical Sciences, Kerman, Iran; 4 Social Determinants of Health Research Center, Institute for Futures Studies in Health, Kerman University of Medical Sciences, Kerman, Iran; 5 Vice-President, Research and Leadership Development, The Canadian College of Health Leaders, Ottawa, Ontario, Canada; 6 Associate, University of Cambridge Judge Business School, Cambridge, United Kingdom; 7 Adjunct professor, Telfer School of Management, University of Ottawa, Ottawa, Canada; 8 Modeling in Health Research Center, Institute for Futures Studies in Health, Kerman University of Medical Sciences, Kerman, Iran; 9 Infectious Diseases Specialist, Clinical HIV/AIDS Fellowship, National HIV/AIDS Care and Treatment Advisor, Ministry of Health, Tehran, Iran; 10 Department of Community and Family Medicine, Faculty of Medicine, Iran University of Medical Sciences, Tehran, Iran; Pan American Health Organization, UNITED STATES OF AMERICA

## Abstract

**Background:**

Integrating clinical programs and services is a cost-effective approach that can improve health and system outcomes. This review aimed to provide an overview of the benefits and challenges of integrated programs for HIV, hepatitis B and C, and STI services worldwide and provide recommendations for research and practice.

**Methods:**

This scoping review followed the Preferred Reporting Items for Systematic Reviews and Meta-Analyses extension for Scoping Reviews (PRISMA-ScR) guidelines. We searched electronic databases PubMed, Scopus, and Web of Science until May 2025 to extract relevant studies. Additionally, we reviewed reputable grey literature sources, such as WHO and UNAIDS, and references from included publications for further relevant articles. Studies that had eligible criteria were included. We applied a narrative approach to report the findings through an inductive approach.

**Results:**

Out of 19,516 initially identified studies, 118 were selected. The benefits and challenges of integration were classified into six categories: integrated service delivery, medical information and technology, human resources, health outcomes, collaboration and partnerships, and financial/physical resources. The significant benefits of these classifications include improved health outcomes, cost-effectiveness, enhanced efficiency, prevention of transmission, use of comprehensive care, reduction of time for receiving necessary services, increased knowledge and awareness, and improved cooperation. However, integration has some challenges, including the need for sufficient infrastructure, budget, human resources, and the potential for increased stress and work pressure on employees.

**Conclusions:**

Prioritizing health is key to national development, requiring policies and resources for cost-effective patient and community benefits. This scoping review highlights the feasibility and advantages of integrating services for HIV, hepatitis B and C, and STIs. Our findings strongly support policymakers in prioritizing the planning and implementation of these integrated programs. An evidence-informed integration framework is needed to guide these actions effectively.

## Background

HIV, hepatitis B and C, and sexually transmitted infections (STIs) continue to affect populations globally. Although a variety of viruses are responsible for these infections, their modes of transmission, such as unprotected sexual contact, exposure to blood, and sharing of needles, and the populations most at risk are very similar [1,2]. According to the most recent UNAIDS global report, 39 million people are living with HIV, and 630,000 have died in the past year from AIDS-related illnesses [3]. In 2022, 254 million and 50 million people were living with hepatitis B and hepatitis C, respectively. The number of deaths from viral hepatitis increased from 1.1 million in 2019 to 1.3 million in 2022, with hepatitis B being responsible for 83% of these cases and hepatitis C accounting for 17%. Daily, approximately 3,500 people worldwide die due to hepatitis B and C virus (HBV and HCV) infections [4]. Additionally, over 1 million treatable STIs are acquired globally each year among individuals aged 15–49, the majority of which are asymptomatic [5].

Co-infection among these infections is common [6,7]. This co-infection further exacerbates liver disease, morbidity, and mortality in people living with HIV (PLWH), particularly if undiagnosed and untreated [8]. Similarly, STIs can significantly impact sexual and reproductive health, contribute to infertility, malignancies, and pregnancy complications, and can increase susceptibility to HIV transmission [5]. Those in resource-limited settings with limited access to diagnosis and medical care are at higher risk of coinfection [9].

Due to the similarity of the transmission, most at-risk populations [2], and also the high prevalence of co-infection of HIV, HCV, HBV, and STIs, integration of services of these infections has been recommended and implemented in some countries [10–12]. In many health facilities, services for HIV, hepatitis B and C, and other STIs are provided through a combination of specialized clinics and integrated care programs. These services are often delivered by multidisciplinary teams that include doctors, nurses, counselors, and laboratory technicians [13]. In some countries, integrated services are offered at the primary care level [14] in others, specialized treatment centers manage the care of patients with these infections [15]. Integrating care and services represents a significant opportunity for preventing and managing these infections, as well as for ending the epidemics [16].

The 2022–2030 World Health Organization (WHO) Global Health Sector Strategies (GHSS) propose a unified vision to end epidemics and advance universal health coverage, primary health care, and health security. The aim is for every person to be able to access high-quality, evidence-based, and people-centered health services. The GHSS specifically targets ending the AIDS, hepatitis B, and C, and STIs epidemics by 2030 [17]. The Strategies recognize that the burden and distribution of HIV, hepatitis B and C, and STIs vary across countries. Consequently, it is essential to adapt responses to align with each region’s specific epidemiological and health system contexts.

Integration, defined as a shift from fragmented and episodic services towards coordinated and seamless two or more services or diseases, offers substantial benefits [18,19]. Integrating HIV, hepatitis B and C, and STI prevention, diagnosis, care, and treatment programs and services can assist health system planners and insurance organizations in allocating resources, making relevant decisions, and improving health outcomes. Therefore, this comprehensive scoping review aimed to provide an overview of integrated HIV, hepatitis B and C, and STI programs and services worldwide. This study aimed to highlight successful interventions and case studies, identify gaps and challenges, and explore emerging developments in the prevention, diagnosis, care, and treatment of HIV, hepatitis B and C, and STIs, while also outlining key considerations for future implementation, which can help in developing a foundational framework.

## Methods

This scoping review followed the Preferred Reporting Items for Systematic Reviews and Meta-Analyses extension for Scoping Reviews (PRISMA-ScR) guidelines [20] and also used Arksey and O’Malley’s methodological framework [21], which consists of five stages. These stages are: [1] identifying the research question, [2] identifying relevant studies, [3] study selection, [4] charting the data, and [5] collating, summarizing, and reporting the results.

### Stage 1: Identifying the initial research questions

This scoping review focused on the benefits and challenges of integrating care for HIV, hepatitis B and C, and STIs within healthcare systems.

### Stage 2: Identifying relevant studies

A systematic search was conducted using electronic databases PubMed, Scopus, and Web of Science until May 2025 with no time or language restrictions to extract relevant papers. We used Medical Subject Headings (MeSH) terms and ‘all fields’ terms, including four core concepts: (HIV), (hepatitis), (sexually transmitted infections), and (healthcare integration). (see Table S1 in **supplementary information**

for the entire search strategy for all databases). We also searched gray literature sources, specifically the official websites of WHO, UNAIDS, and CDC, using the same keywords and inclusion criteria. Additionally, we conducted a search in Google Scholar, where for each distinct keyword combination (search query), we screened the first ten pages of results (equivalent to 100 records per query). No language or date filters were applied at this stage to ensure a comprehensive search. Finally, we used citation chaining (backward by one step) to review the reference lists of the included research for relevant studies that were not found during our original search. We used EndNote X21 software to manage the extracted documents, remove duplicates, and save details for review.

### Stage 3: Study selection

**Fig 1:**
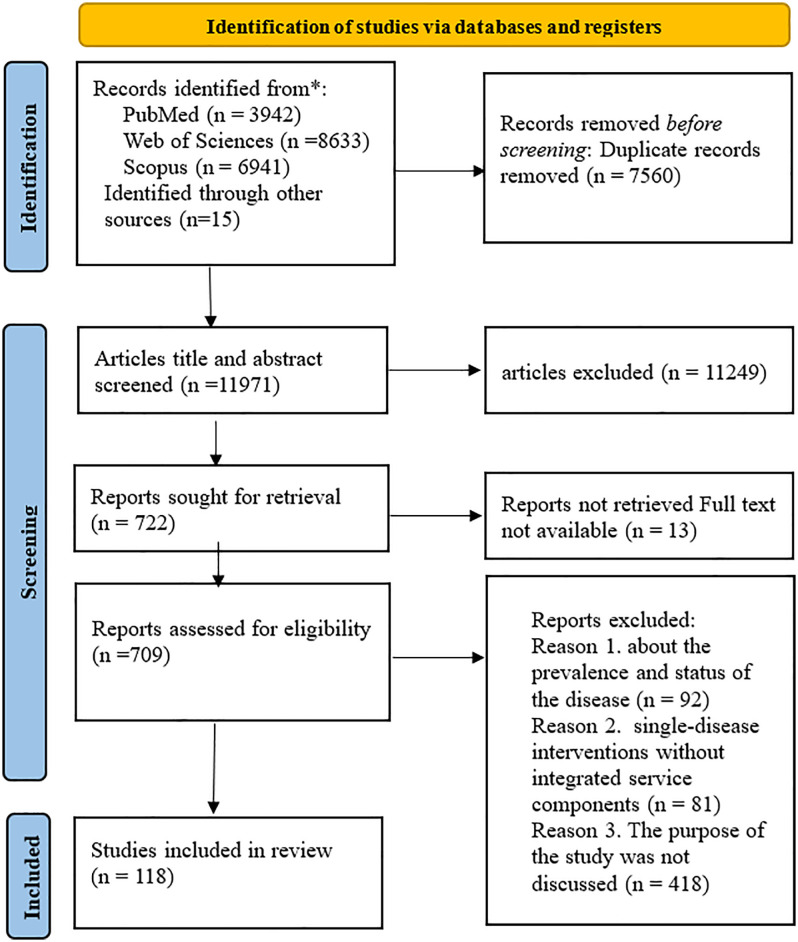
PRISMA flowchart for selection of documents about the benefits and challenges of integrating services of HIV, hepatitis B and C, and STIs.

The initial search yielded 19,516 documents. After removing duplicates, we reviewed the remaining 11,956 documents based on their titles and abstracts. Of these, 11,249 articles were excluded, and 707 articles advanced to the full-text review stage. We identified an additional 15 documents through the bibliography screening of the included documents. After reviewing the full texts thoroughly according to the study’s purpose and criteria, we identified 118 relevant articles. All steps were conducted by two independent reviewers (PJA and ZA). In the event of disagreement between two reviewers, these issues were discussed among the authors until they were resolved. Fig 1 illustrates the study selection process using the Preferred Reporting Items for Systematic reviews and Meta-Analyses (PRISMA) flowchart.

### Inclusion and exclusion criteria

#### Inclusion criteria.

We included qualitative and quantitative studies and mixed-methods studies related to integrated programs and services, encompassing experimental and observational research, reports, program evaluations, editorials or letters, and viewpoints. Eligible studies addressed HIV, hepatitis B and C, and STIs, focusing on prevention, diagnostic testing, referral to care, treatment, adherence, viral suppression, and reported cost outcomes, such as health outcomes and cost-effectiveness. The documents included in this study integrated at least two of the target infections: HIV, HBV/HCV, and STIs. While the primary emphasis was on the simultaneous integration of HIV, HBV/HCV, and STIs, we also included studies that addressed the integration of any two or more of these conditions. These studies focused on integrated services and other forms of integration, such as those involving healthcare providers, infrastructure, resources, monitoring, evaluation, and supply chain management, and covered various types of facilities, including hospitals and community-level services.

#### Exclusion criteria.

We excluded studies that did not involve integrated services for HIV, hepatitis B and C, and STIs, or those that did not report corresponding outcomes. We also excluded non-peer-reviewed literature, study protocols, books, dissertations, and journal articles without full-text access. Additionally, reviews were excluded, as single studies were included. We excluded case reports and case series, as they focused on individual-level observations and did not contribute to understanding system-level integration or programmatic insights into integrated service delivery, which was the core focus of our study. We excluded clinical trials that focused solely on the clinical efficacy or safety of a specific intervention for a single infection without incorporating integrated service delivery components involving at least two of the targeted conditions, unless they explicitly addressed integrated service delivery models.

### Stage 4: Data charting

We recorded relevant extracted information from the included documents in a Microsoft Word table. Details recorded in this table included: the first author, year of publication, country, study population and size, study or program objectives and aims, study design, program details, reported program outcomes, and the benefits and challenges of integration (see S2 Table).

### Stage 5: Collating, summarizing, and reporting the results

We employed a narrative approach, a commonly used method in scoping reviews to summarize findings from heterogeneous studies. A narrative approach was used to analyze and organize the extracted data from the included studies. After thoroughly reviewing the content of each study, relevant information regarding the integration of HIV, hepatitis B, and C, and STIs services was systematically extracted. The data were inductively coded and grouped into six major categories that highlighted the key characteristics across studies. The initial inductive coding was performed by the first author PJA. Following the initial coding, the preliminary codes were shared with the other research team. Colleagues provided their feedback and perspectives, which allowed for the refinement and piloting of the coding framework through iterative discussion. Any disagreements or discrepancies that arose during this collaborative process were resolved through discussion among the team members until a consensus was reached. This repeated process was important in developing and finalizing the categories and sub-categories. These categories were systematically coded and organized according to the key domains of integration (Table 2).

### Ethics approval

This research has been registered with the Ethics Committee of Kerman University of Medical Sciences (Approval ID IR.KMU.REC.1402.352).

## Results

Of the initial yield of 19,516 extracted articles, 118 met the inclusion criteria following stages of title, abstract, and full-text review. Most were qualitative studies (50.0%) set in high-income economies (60.8%), and were published after 2015 (61.8%). In terms of the composition of integrated programs, the most common were HIV and hepatitis B and C (n = 41, 34.8%) and HIV and STIs (n = 36, 30.5%). (Table 1)

**Table 1:** Characteristics of the extracted documents about integrated HIV, hepatitis B and C, and sexually transmitted infections programs and services.

Output	N	Percent	Output	N	Percent
**Years of publications**			**Integrated diseases**		
< 2000	1	0.8	HIV, hepatitis B and C and STIs	31	26.2
2000-2004	5	4.2	HIV and hepatitis B and C	41	34.8
2005-2009	20	17.0	HIV and STIs	36	30.5
2010-2014	19	16.2	hepatitis B and C and STIs	8	6.8
2015-2019	31	26.2	STIs	2	1.7
≥ 2020	42	35.6	**Type of study**		
Countries by economy*			Economic evaluation	5	4.2
High income	65	60.8	Mix Method	6	5.1
Upper-middle-income	22	20.6	Quantitative study	16	13.6
Lower-middle-income	16	14.9	Qualitative study	59	50.0
Low-income	4	3.7	Viewpoint, perspective	5	4.2
			Report, principle, program	21	17.8
			Editorial, correspondence	6	5.1

*Total is less than 118, as some documents, such as viewpoints and editorials were not country-specific

Following our narrative analysis, we categorized the findings of reported benefits and challenges of integrated HIV, hepatitis B and C, and STIs programs and services. The categories are: 1) integrated service delivery, 2) medical information and technology, 3) human resources, 4) health outcome, 5) collaboration and partnership, and 6) financial/physical resources (Table 2).

**Table 2:** Benefits and challenges of integrating programs and services of HIV, hepatitis B, C, and sexually transmitted infections according to the narrative approach.

Category	Benefits	Challenges
**Integrated service delivery**	• Reduced time and increased convenience for receiving necessary services [10,14,22–29] • Feasibility [11,30–35] • Acceptability [14,31,36–45] • Comprehensive and holistic care [11,13,16,23,24,27,29,31,33,34,46–62] • Increased coverage of screening [26–28,30,33,39,40,43,50–52, 63–84] • Out‑of‑pocket [11,23,26,36,46,85,86] • Reduced stigma and discrimination [27,42,87,88]	• Privacy challenges [14] • Reduced service quality [11,14,87] • Low community participation [27,53,61] • Increased stigma and discrimination [27,36,71,87–90] • Organizational structural and cultural challenges [38,42,47] • Language barriers [14,29] • Negative mental health problems [12,72,78]
**Medical information and technology**	• Improving the integration of program data systems [22,27,31,67] • Sharing of technical expertise and equipment [23,24,27,28,72,91]	• Data and information system challenges [29,32,36,40,92–95] • Absence of an established integration strategy or framework [55,95,96]
**Human resources**	• Increased knowledge and awareness [25,33,38,56,87,88,97–101] • Improved multi-tasking and better cross-training [15,38,71,88,91]	• Inadequate staff and population knowledge [14,27,29,42,47,102] • Lack of training [24,36,38,42,84,90,103] • Increase workload [11,27,36,59,72,104]
**Health outcomes**	• Improved efficiency [22,46,49,55,69,102,104,105] • Cost-effectiveness [10,12,23,26,36,46,54,55,85,89,92,106–113] • Transmission prevention [10–12,26,27,29,30,32,50,51,54,63,67,85,88,92,93,95,97,99,100,106,108,114–125]	
**Collaboration and partnership**	• Increasing collaboration and building new partnerships [11,22,47,55,56,91,92,126] • Creating mutual trust and sustainable connections [14,103,122]	• Low level of experience of collaboration [13,25,47,48,95,127]
**Financial/physical resources**	• Sharing of resources and equipment [23,24,27,28,72,91]	• Economic and resources constraints [14,24,25,27,32,33,38,41,44,69,71,92,94,95,97,104,121,122,128–131]

### 1) Integrated service delivery.

This category encompasses factors related to service mechanisms, processes, and interactions among them. The identified factors from the reviewed studies are detailed below.

### Benefits

#### 1. Reduced time and increased convenience for receiving necessary services.

Ten studies in total indicated that integrating services for these diseases significantly reduces clients’ time in obtaining care and other necessary services such as testing and vaccination. By consolidating services at a single location, clients avoid multiple visits to different sites, thus saving time [10,14,22–29].

#### 2. Feasibility.

The feasibility of integrating services has been widely discussed. In Vietnam, for example, policymakers reported being optimistic about the health benefits of integration, especially for preventing mother-to-child transmission (MTCT) of HIV, HCV, and syphilis [30]. In Western Uganda, integrating HIV and hepatitis B services is viewed as beneficial for both patients and healthcare providers [11,31,32]. Furthermore, integrating hepatitis C education and screening into existing primary care programs has been proposed to enhance disease management [33,34]. A study conducted in South Africa showed that interventions on HIV integrated with sexual health were feasible and popular, especially for young people [35].

#### 3. Acceptability.

Several countries reported positive experiences and acceptance of integrated services [14,31,36–43,45]. For instance, in Vietnam, key at-risk populations, including people who inject drugs (PWID), non-injecting drug users (DU), men who have sex with men (MSM), female sex workers (FSW), and transgender women (TGW), have shown high acceptance levels [44].

#### 4. Comprehensive and holistic care.

The integrated approach offers a comprehensive package that includes prevention, diagnosis, treatment, and care [13,24,27,29,33,34,46–52,60–62]. Studies from various countries highlighted that this approach reduces the number of patient visits, accelerates diagnosis [11,23,31,53,54], and that, by providing the same diagnostic testing and treatment to those either coinfected with HIV and HCV or at higher risk, better addresses their health needs [16,55–58]. For example, in New York City, clients visiting clinics for hepatitis vaccines or screening were also checked for HIV and STIs, resulting in benefits from STIs/HIV examinations, testing, treatment, and referrals they may not have received otherwise [59].

#### 5. Increased coverage of screening.

Enhanced screening procedures across infections are frequently cited as a significant benefit. By screening individuals for HIV, viral hepatitis, and STIs concurrently, enhanced overall screening leads to earlier diagnosis and initiation of appropriate treatment, thereby reducing the risk of disease transmission [26–28,30,33,39,40,43,50–52, 63–84].

#### 6. Out-of-pocket.

Integration of services often reduces costs for low-income and key populations, since the majority of the people who want to receive these services are people from the lower socioeconomic class of society, who pay less money or receive their services for free [23,26,36,46,85,86]. In many cases, services are provided at no cost. For instance, China could provide free testing for these three infections with integration [11].

#### 7. Reduced stigma and discrimination.

Stigma is the negative attitude towards people that causes them to be deprived of services or a condition. Integration of services has been associated with reduced stigma and discrimination. Studies from Uganda [87], China [88], and Mississippi (US) [42] highlighted that integration can reduce stigma and discrimination across various levels. Additionally, research in Kenya indicated that the stigma among men was reduced [27].

### Challenges

Despite these benefits, several challenges associated with service delivery integration have been identified.

#### 1. Privacy challenges.

Individuals with HIV and hepatitis, who face significant stigma, often express privacy concerns. Healthcare providers confirm that patients’ primary worries relate to privacy and confidentiality. This anxiety is largely driven by the fear that utilizing their insurance or seeking medical care might inadvertently disclose conditions like HIV or a history of injection drug use, potentially leading to negative repercussions in their employment. Such fears can act as a significant barrier to accessing healthcare [14].

#### 2. Reduced service quality.

Integration of services for these diseases can sometimes decrease service quality. Studies from Uganda and China have reported reduced service quality [11,87]. A survey across several US locations found that integrated services sometimes led to limited testing services [14].

#### 3. Low community participation.

Some healthcare providers have noted that integration may exacerbate stigma, as some patients with STIs might be reluctant to be associated with HIV infection through the integration of STIs and HIV services. This led to declining community participation [27] and a lack of engagement in healthcare [53,61].

#### 4. Increased stigma and discrimination.

Contrary to the benefits, some studies have reported an increase in stigma and discrimination [27,36,71,87,90]. For example, research on FSWs identified stigma and discrimination as critical structural issues that deterred vulnerable women from seeking treatment [88] because of a criminological view of high-risk behaviors, which has been mentioned [89].

#### 5. Organizational structural and cultural challenges.

Despite the potential benefits of integrated programs and services, organizational structural and cultural barriers within and among partner organizations can obstruct or inhibit implementation and success. For example, in the UK, existing leadership and management structures and cultures are reportedly not conducive to integration [38]. Similarly, studies in the US demonstrated the need for training and education to facilitate the culture shifts within and among hospitals and public health organizations that is required to integrate programs successfully [42,47].

#### 6. Language barriers.

This includes language, since one study identified communication complications when providers and recipients of services lacked common languages [14]. In the Koboko district of Uganda, for example, patients speak various languages, such as, Lugbara, Kakwa, Lingala, and Arabic, and when healthcare providers have lacked fluency in these languages or interpreters were not available, healthcare workers provided services without appropriate or adequate information [29].

#### 7. Negative mental health problems.

Both young people and healthcare workers expressed concerns about the potential adverse mental health problems of unexpected test results. They emphasized the importance of pre-and post-test counseling and seamless linkage to care for patients [78].There is a high comorbidity of mental health or substance use issues [12] and increased stress among counselors due to the number of positive HCV test results given to clients [72].

#### 2). Medical information and technology.

This category relates to disease management information systems and the use and sharing of technology. The benefits and challenges are detailed below.

### Benefits

#### 1. Improving the integration of program data systems.

Studies conducted in Florida and Hawaii, USA, showed that integration improved data entry quality into the system [22]. In Uganda, the integration of services improved the reporting field [31]. In Kenya, it provided an opportunity to use the existing HIV care system for HBV care [27]. In Gaborone, Botswana, all participants could offer adequate self-collected specimens, were informed of their results, and were linked to treatment if needed [67].

#### 2. Sharing of technical expertise and equipment.

The integration approach uses existing capacity better by sharing facilities such as laboratories, medical devices, testing tools, and personnel [23,24,27,28,72,91].

### Challenges

#### 1. Data and information system challenges.

In several countries, patient data is collected separately for each disease, resulting in disparate data systems and nonsystematic documentation [36,92]. Furthermore, test results are not always routinely documented, and there can be documentation errors or legibility issues with hand-written notes, such as those by physicians. A health management information system is needed to capture HCV data, since without one, these defects make integrated assessment and data collection nearly impossible [29,40,93–95]. These issues further hinder the ability of disease intervention specialists to track outreach index cases and their partners [36]. For example, it was not feasible to ascertain the full extent of the HBV vaccine series completion due to the unavailability of a system for individual client data tracking [32].

#### 2. Absence of an established integration strategy or framework.

Many countries undergoing integration have reported the need for a credible strategy and framework to guide integration and define tasks, the absence of which presents significant challenges [55,95]. Even in British Columbia, Canada, although healthcare providers had access to provincial guidelines, one study reported that they were not generally used [96].

#### 3) Human resources.

This category outlines the benefits and challenges related to staff and health workers.

### Benefits

#### 1. Increased knowledge and awareness.

Integration programs have increased patients’ and staff’s knowledge and awareness of diseases, their transmission, prevention, and treatment [33,38,56,87,88,97–101], positively impacting behaviors and outcomes [25].

#### 2. Improved multi-tasking and better cross-training.

Training for healthcare staff and physicians in integration typically clearly defines the roles of clients, healthcare staff, and physicians regarding of diagnostic, screening, treatment, and follow-up care, which is important for effective virus management [15,88]. Similarly, cross-training for staff improves time management, awareness, and acceptance [38,71], helping staff find solutions to problems more efficiently [91].

### Challenges

#### 1. Inadequate staff and population knowledge.

Despite the increased knowledge from integration programs, several studies have identified insufficient knowledge about HBV, HCV, and STI management among health workers in Kenya, France, Uganda, and the United States [14,27,29,102]. More specifically, healthcare providers in non-urban areas tend to lack knowledge about HIV and STIs management [42]. Patients in the Netherlands and the United States also show a lack of knowledge, particularly regarding sexual health, which leads to increased virus transmission [14,47].

#### 2. Lack of training.

In several countries, there is an identified need for improving staff training. Developing a continuous education system in a new, culturally relevant format is necessary. Attention is needed for training regarding STIs and people with experience and expertise in one disease should also be trained about other diseases [24,36,38,42,84,90,103].

#### 3. Increase workload.

Integrating services often increases employees’ workloads [27,36,59,104], leading to higher stress levels [72]. A study conducted in China showed that inadequate staffing in integration experiences exacerbates this issue, further increasing employees’ stress and workloads [11].

#### 4) Health outcomes.

This category examines the health consequences of integration on health systems and patients, based on the available evidence, which mainly reported benefits. No specific challenges were mentioned in the studies included.

### Benefits

#### 1. Improved efficiency.

Integration within the health system enhances efficiency [49,55,69,102,104,105], particularly in areas with constrained resources [22]. A study also supports the claim that integration improves the efficiency of service delivery systems [46].

#### 2. Cost-effectiveness.

Integration programs demonstrate cost savings by sharing equipment, materials, staff, or services [23]. For instance, integrating pre-exposure prophylaxis of HIV with hepatitis C treatments is cost-effective for PWID, and repeated tests are cost-effective for high-risk groups [89]. Additionally, HIV screening programs combined with syphilis screening have proven to be more cost-effective than HIV screening alone [106]. Cost-effectiveness is evident for both clients and health systems across various countries [10,12,26,36,46,54,55,85,92,107–113].

#### 3. Transmission prevention.

Integrated services facilitate the delivery of preventive measures [95], including vaccination, HIV and STI prevention services, and simultaneous testing for HIV, HBV, HCV, and STIs at one center [11,29,32,50,97,108,114,115]. This approach has led to increased screening rates [12,63]. One study showed an initial increase in the number of positive cases detected following the introduction of an integrated program [63] and a subsequent decline in the number of positive tests over the following years [116]. Simultaneous testing provides critical information to individuals about their condition, helping prevent the transmission of diseases to partners, especially in high-risk groups [93]. Integration of services also prevents MTCT of HIV, HBV, HCV, and syphilis [30,51,117,118], as well as adverse outcomes during pregnancy [54,106]. For example, in Cambodia, an integrated approach reduced MTCT rates for HIV from 6.6% to 6.1%, HBV from 14.1% to 3.4%, and syphilis from 9.4% to 4.6% [10]. Prevention services available at integrated care centers, such as free condoms and other incentives, encourage individuals who previously did not access services to seek and utilize them [119]. Studies across various countries demonstrate the significant impact of preventive services on disease transmission, highlighting the success of the integration [26,27,67,85,88,92,99,100,120–125].

#### 5) Collaboration and partnership.

This section outlines the benefits and challenges of intra-organizational collaboration between employees or departments and extra-organizational collaboration among organizations.

### Benefits

#### 1. Increasing collaboration and building new partnerships.

Collaboration during the integration process enhances immunization activities for high-risk groups and increases system flexibility in budget allocation, creating a more conducive environment for cooperation [22]. This is particularly true for disease-related programs and their control [91,92,126] or directing patients regarding treatment planning and time [56]. Furthermore, the initiative will facilitate inter-center collaboration with the Center for Disease Control and hospitals, enhancing intersectoral cooperation to achieve the WHO’s objective [11,47]. In Massachusetts, mergers have improved communication and collaboration among customers, service providers, government, and financial institutions, offering potential cost savings and broader service delivery [55].

#### 2. Creating mutual trust and sustainable connections.

Supportive and honest relationships between service providers and patients enhance cooperation, build trust, and ensure timely treatments, particularly for sex workers [14,103,122].

### Challenges

#### 1. Low level of experience in collaboration.

Countries with integration experiences have identified several obstacles to cooperation, including low levels of cooperation in legal and equipment matters [127], poor coordination between hospitals and health services [47], insufficient collaboration between service providers and caregivers [13,25], lack of referral and clear communication systems [95], and inadequate communication between primary care and clinics [48]. This latter issue was particularly problematic, as it often involved a paper-based system and receipts, which made it difficult for patients to have a proper treatment regimen.

#### 6) Financial/physical resources.

This category encompasses the equipment, infrastructure, and financial and physical resources utilized and allocated concurrently in service integration, presenting the benefits and challenges.

### Benefits

#### 1. Sharing of resources and equipment.

Pooling resources, including equipment, consumables, services, and maintenance, reduces costs and enables healthcare providers to deliver a more appropriate health response [23,27,28,72]. Joint use of laboratory and care services capacities and administering STI, HIV, and HCV tests concomitantly increases the potential number of diagnoses. It identifies outbreaks more rapidly, enabling quicker response interventions [24]. For instance, integrating services in Atlanta (US) improved the sharing of technical and expert resources and tools [91].

### Challenges

#### 1. Economic and resource constraints.

Fifteen studies in total have identified significant barriers to integration, including a lack of resources and inadequate financial support [24,25,33,38,44,69,71,92,94,95,104,121,122,128,129]. These constraints impact various integration aspects, including patient care and follow-up [32], hindering the implementation of elimination strategies, vaccine procurement, and prevention measures [41,97,130,131], as well as the availability of STI drugs and diagnostics, including test quantities [14,27]. They also impede the treatment of HCV infection [33].

## Discussion

This scoping review has identified that integrated programs and services for HIV, hepatitis B and C, and STIs can positively influence several health outcomes related to the prevention, detection, and treatment of viruses. Additionally, they are more efficient and cost-effective for both patients and healthcare providers. Along with these benefits, several challenges exist, the most common being funding and resource constraints, which need to be addressed for successful program integration. Our narrative analysis identified six categories of benefits and challenges linked to integrating HIV, hepatitis B and C, and STI programs. These categories are: integrated service delivery, medical information and technology, human resources, health outcomes, collaboration and partnership, and financial/physical resources. Each category contains a few sub-categories of benefits and challenges.

Benefits of integrated service delivery include time savings, feasibility, increased acceptance, comprehensive services, increased screening coverage, reduced out-of-pocket expenses for recipients, and reduced stigma and discrimination. Performing all tests in one location saves time, especially for individuals in high-risk groups; instead of visiting multiple locations, they can complete all tests in one visit [23]. Establishing a centralized facility offering an extensive range of services within the community enables effective communication and the delivery of comprehensive patient care [132]. In other words, integrating services makes it easier for people to get more services when they refer to sexual and reproductive health clinics. It also increases people’s acceptance of HIV testing or Pre-exposure prophylaxis (PrEP) treatment [133–135]. Studies have shown that integration increases screening coverage, as well as simultaneous HIV and hepatitis C and B testing in prisons, leads to faster identification [136,137], which can lead to proper treatment [138]. Integration can promote equity by reducing structural barriers and improving the reach of marginalized or underserved groups [84]. It also enhances access by enabling individuals to obtain multiple forms of support within a single, coordinated framework, thereby reducing logistical and financial obstacles [102]. Furthermore, integrated models can mitigate stigma by normalizing help-seeking behaviors, particularly when sensitive services (e.g., mental health or HIV care) are embedded within general health or social services. This approach allows individuals to engage with care without being singled out or labeled [87]. On the other hand, HIV, viral hepatitis, and STIs have an asymptomatic phase [139]. If not detected early, they can lead to irreversible complications. These include various types of cancer and infertility [140]. A study conducted among PWID found that integrating HIV, HCV, tuberculosis, needle and syringe exchange services for them improved their HIV-related outcomes [141]. Some key populations, such as PWID, were reluctant to receive HIV or HCV services due to financial problems [142]. By integrating services, the cost was minimized for them [143]. The expansion of service delivery in PHC is further supported by integrated diagnostic platforms, such as multiplex RDTs and networks, which benefit from tiered planning, specimen referral, and LIS interoperability. Additionally, WHO-approved self-tests for HIV, syphilis, and HCV are significant advancements for improving service accessibility and individual empowerment [144,145].

However, these benefits come with significant challenges, such as maintaining patient privacy, particularly for those infected with HIV and hepatitis. Additionally, the quality of services often needs to improve, patient participation in some areas is limited, and stigma and discrimination persist. Organizational structural and cultural challenges, multiple languages, and potential negative consequences of knowing infection status, such as anxiety and depression, further complicate service delivery. Service workers reported that infected patients, especially those from key populations, were discouraged from attending HIV and STI clinics because of concerns about privacy and loss of confidentiality [146]. A study has shown that as the number of referrals increases, the quality of services decreases if the necessary infrastructure does not exist [147]. Stigma and discrimination are major obstacles to accessing health and prevention services, exacerbated by cultural or socioeconomic differences [148,149]. Stigma can occur at all levels of service delivery and has a significant impact on the delivery of services to people living with viral hepatitis and HIV [150]. PWID are among those prioritized for these services because of their risky behavior, but they did not seek services because of stigma, discrimination, and lack of motivation due to depression [142]. There appears to be a controversy regarding the impact of service integration on stigma and discrimination. While some studies reported a reduction in stigma due to normalization and broader access, others highlighted concerns about increased stigma resulting from reduced confidentiality. These conflicting findings highlight the complexity of this issue and indicate the need for more in-depth and further studies. As Alege et al. highlighted, Language barriers remain a significant issue affecting service delivery [29].

The benefits of medical information and technology include improved integration of program data systems, facilitating more accurate data collection, enhancing communication for patient follow-up, and scheduling treatment or prevention services. A study in Uganda showed that integrating HIV and hepatitis B and C services improved the quality of information recording and fostered positive relationships among sectors [29]. However, challenges remain, including fragmented data and the lack of integrated, systematic registration systems, which result in inadequate patient monitoring. One major drawback is the absence or non-use of comprehensive guidelines for integration in some countries. Conflicting experiences indicate that further studies are needed to draw definitive conclusions. Engage in developing integrated electronic health record systems that enable tracking patient data across multiple conditions, resulting in timely and accurate follow-up and treatment planning [53].

In the human resources category, integration has been found to enhance the knowledge base of both patients and service providers, facilitate their education and training, and enable them to perform a range of tasks proficiently. Studies have shown that the integration of services can be beneficial for young people, especially in terms of prevention, which has increased knowledge and modified sexual behaviors such as condom use [133,151]. However, employee knowledge is inadequate in some countries, indicating that they must receive sufficient training to prevent, detect, and manage these diseases effectively. Increased workload attributed to providing integrated services has led to psychological distress and job turnover among personnel. Other studies have found insufficient knowledge among staff about HIV and hepatitis C [152,153]. Alege et al. noted that a lack of sufficient staff increases the workload [29]. Mohanty et al. highlighted the need for greater awareness regarding HBV among patients and inadequate knowledge among service providers [154]. Given the conflicting evidence, further investigation is needed to draw definitive conclusions on the impact of integration on employee knowledge and training. Emphasis on regular training and development programs for healthcare providers to help them improve their knowledge and abilities in managing integrated care for HIV, hepatitis B and C, and STIs.

Service integration has demonstrated benefits with no discernible disadvantages regarding service-related health outcomes. Benefits include increased system efficiency, cost-effectiveness, and prevention of MTCT or between sexual partners. Nkulu-Kalengayi et al. demonstrated that system-level interventions, such as HBV and chlamydia vaccinations and HIV and HBV screening programs, effectively reduce disease transmission [155]. Implementing hepatitis B vaccination programs is a cost-effective strategy for preventing costs to the health system and infected patients [156]. The integration has also been shown to be cost-effective in preventing MTCT of HIV, hepatitis B, and syphilis [10]. Given its cost-effectiveness and proven role in preventing transmission, integrated service delivery can be prioritized in national health strategies.

Collaboration and partnership benefits include increased cooperation and collaboration between clients and service providers, facilitated by increased honesty and trust. This collaboration facilitates a more equitable distribution of services in countries with constrained resources, particularly for vulnerable and marginalized populations [157]. While integrating services is said to increase cooperation in some countries, it is reportedly low in some regions. The level of cooperation varies according to the resources or infrastructure available in different countries. To improve the quality and convenience of services provided to patients, it is recommended that more intersectoral and inter-organizational cooperation be established between patients and service providers.

In the financial and physical resources category, benefits include sharing resources and equipment and utilizing capacities, personnel, and equipment to provide services to patients with different diseases. However, many countries lack adequate financial resources, equipment, and other essential materials during integration. Increased patient numbers and the demand for services exacerbate these resource shortages. Concerns include insufficient diagnostic kits and inadequate supplies of drugs for STIs. Alege et al. reported a need for more financial resources for continued patient monitoring and testing kits [29]. Mohanty et al. highlighted a lack of HBV vaccines and funding for preparing HBV vaccines, especially for adults [154]. Financial problems and resource constraints impact service quality. Providing people with proper quality services is impossible, leading to a lack of referrals and discouraging ongoing patient treatment. Thus, each country should strive to attract financial funds at the governmental and international levels based on its capacity to integrate services successfully.

### Study strengths and limitations

This scoping review is the first to provide comprehensive evidence of the integration of HIV, hepatitis B and C, and STIs services. It has provided benefits and challenges of integration from two perspectives that should have been considered in previous studies. This can assist policymakers and planners of the health system and the field of service delivery in considering the integration of communicable disease services to maximize benefits and minimize challenges.

This study has four limitations. First, it is possible that the selected literature does not represent all relevant studies on the integration of HIV, hepatitis B and C, and STIs, as some studies may have been published are not available in the databases that we searched. To mitigate this, we conducted a comprehensive search of several major databases. We included gray literature, specifically the official websites of WHO, UNAIDS, and CDC, using the same keywords and inclusion criteria to capture relevant documents and screening the first ten pages (n = 100 results in total) of Google Scholar. Finally, we used citation chaining (backward by one step) to review the reference lists of the included research for relevant studies that were not found during our original search. Second, the quality and setting of the included studies vary, complicating the synthesis of findings due to differences in healthcare systems, cultural contexts, and implementation strategies. Regarding heterogeneity in study quality and setting, we recognize that differences in health care systems and cultural contexts may complicate synthesis. However, we believe that this diversity also enriches our understanding of how integration is approached in different settings. Third, the dynamics of healthcare systems are in continuous flux, necessitating strategies evolve and adapt alongside developments in the context. Our discussion reflects this by emphasizing the need for adaptive and context-sensitive integration strategies. Fourth, many countries may have experiences with service integration that were never publicly documented, making them inaccessible for this review. This is an inherent limitation of review studies, as they rely on available and published sources.

## Conclusions

The scoping review reveals that HIV, hepatitis B and C, and STIs have characteristics in common and share common methods of prevention, diagnosis, and treatment. This makes the integration of these diseases feasible. However, the process presents both significant benefits and challenges. Key benefits include facilitating patient healthcare services, disease transmission prevention, cost-effectiveness, and increased efficiency. On the other hand, significant obstacles include insufficient funding, lack of inter-organizational and national collaboration, and inadequate resources and expertise. Addressing these obstacles is important for the successful implementation of integrated programs and for maximizing benefits to patients and the health system. Our findings indicate that policymakers should consider prioritizing the development of initiatives that support the integration of these disease services, recognizing the potential for a more streamlined and effective public health approach.

## Supporting information

S1 Table Search strategy in all databases (PubMed, Scopus, and Web of Science). (DOCX)

S2 Table Summary of characteristics of the documents included in the scoping review (n = 118). (DOCX)

## Abbreviations

STI: sexually transmitted infection
HBV: hepatitis B virus
HCV: hepatitis C virus
PLWH: people living with HIV
WHO: World Health Organization
GHSS: Global Health Sector Strategies
PRISMA: Preferred Reporting Items for Systematic reviews and Meta-Analyses
MTCT: mother-to-child transmission
PWID: people who inject drugs
DU: drug users
MSM: men who have sex with men
FSW: female sex workers
TGW: transgender women
PrEP: Pre-exposure prophylaxis
