# Supplementary material for: Integration of HIV, Hepatitis B, and C, and sexually transmitted infections services: A scoping review of the benefits and challenges
Source: PLoS One. 2026 May 7;21(5):e0348073. doi: 10.1371/journal.pone.0348073 (PMC13152153; doi:10.1371/journal.pone.0348073)
Supplement: S1 Table — (DOCX) [file pone.0348073.s001.docx]

Table S1. Search strategy in all databases (PubMed, Scopus, and Web of Science)

| **Search strategy of PubMed=A OR B OR C OR D** | |
| --- | --- |
| **A:** ("Human Immunodeficiency Virus"[Title/Abstract] OR AIDS[Title/Abstract] OR HIV[Title/Abstract] OR "Acquired immunodeficiency syndrome"[Title/Abstract]) AND ("sexually transmitted infections"[Title/Abstract] OR STD[Title/Abstract] OR STDs[Title/Abstract] OR "sexually transmitted diseases"[Title/Abstract] OR "Venereal Diseases"[Title/Abstract] OR STIs[Title/Abstract] OR STI[Title/Abstract]) AND (integrat*[Title/Abstract] OR pooling[Title/Abstract] OR merge[Title/Abstract] OR merging[Title/Abstract] OR linkage[Title/Abstract] OR combine[Title/Abstract] OR join[Title/Abstract] OR unified[Title/Abstract] OR unify[Title/Abstract] OR union[Title/Abstract] OR consolidate[Title/Abstract] OR incorporat*[Title/Abstract]) | (HIV& STI) |
| **B:** ("Human Immunodeficiency Virus"[Title/Abstract] OR AIDS[Title/Abstract] OR HIV[Title/Abstract] OR "Acquired immunodeficiency syndrome"[Title/Abstract]) AND (Hepatitides[Title/Abstract] OR Hepatitis[Title/Abstract]) AND (integrat*[Title/Abstract] OR pooling[Title/Abstract] OR merge[Title/Abstract] OR merging[Title/Abstract] OR linkage[Title/Abstract] OR combine[Title/Abstract] OR join[Title/Abstract] OR unified[Title/Abstract] OR unify[Title/Abstract] OR union[Title/Abstract] OR consolidate[Title/Abstract] OR incorporat*[Title/Abstract]) | (HIV &hepatits) |
| **C:** ("sexually transmitted infections"[Title/Abstract] OR STD[Title/Abstract] OR STDs[Title/Abstract] OR "sexually transmitted diseases"[Title/Abstract] OR "Venereal Diseases"[Title/Abstract] OR STIs[Title/Abstract] OR STI[Title/Abstract]) AND (Hepatitides[Title/Abstract] OR Hepatitis[Title/Abstract]) AND (integrat*[Title/Abstract] OR pooling[Title/Abstract] OR merge[Title/Abstract] OR merging[Title/Abstract] OR linkage[Title/Abstract] OR combine[Title/Abstract] OR join[Title/Abstract] OR unified[Title/Abstract] OR unify[Title/Abstract] OR union[Title/Abstract] OR consolidate[Title/Abstract] OR incorporat*[Title/Abstract]) | (STI&hepatits) |
| **D:** (Hepatitides[Title/Abstract] OR Hepatitis[Title/Abstract]) AND ("Human Immunodeficiency Virus"[Title/Abstract] OR AIDS[Title/Abstract] OR HIV[Title/Abstract] OR "Acquired immunodeficiency syndrome"[Title/Abstract]) AND ("sexually transmitted infections"[Title/Abstract] OR STDs[Title/Abstract] OR STD[Title/Abstract] OR "sexually transmitted diseases"[Title/Abstract] OR "Venereal Diseases"[Title/Abstract] OR STIs[Title/Abstract] OR STI[Title/Abstract]) AND (integrat*[Title/Abstract] OR pooling[Title/Abstract] OR merge[Title/Abstract] OR merging[Title/Abstract] OR linkage[Title/Abstract] OR combine[Title/Abstract] OR join[Title/Abstract] OR unified[Title/Abstract] OR unify[Title/Abstract] OR union[Title/Abstract] OR consolidate[Title/Abstract] OR incorporat*[Title/Abstract]) | (HIV&STI&Hepatits) |
| **Search strategy of Scopus=A OR B OR C OR D** | |
| **A:** ( TITLE-ABS-KEY ( "human immunodeficiency virus" OR aids OR hiv OR "acquired immunodeficiency syndrome" ) AND TITLE-ABS-KEY ( "sexually transmitted infections" OR stds OR std OR "sexually transmitted diseases" OR "venereal diseases" OR stis OR sti ) AND TITLE-ABS-KEY (integrat* OR pooling OR merge OR merging OR linkage OR combine OR join OR unified OR unify OR union OR consolidate OR incorporat* ) ) | (HIV& STI) |
| **B:** ( TITLE-ABS-KEY ( "human immunodeficiency virus" OR aids OR hiv OR "acquired immunodeficiency syndrome" ) AND TITLE-ABS-KEY ( hepatitides OR hepatitis ) AND TITLE-ABS-KEY (integrat* OR pooling OR merge OR merging OR linkage OR combine OR join OR unified OR unify OR union OR consolidate OR incorporat* ) ) | (HIV &hepatits) |
| **C:** ( TITLE-ABS-KEY ( "sexually transmitted infections" OR std OR stds OR "sexually transmitted diseases" OR "venereal diseases" OR stis OR sti ) AND TITLE-ABS-KEY ( hepatitides OR hepatitis ) AND TITLE-ABS-KEY (integrat* OR pooling OR merge OR merging OR linkage OR combine OR join OR unified OR unify OR union OR consolidate OR incorporat* ) ) | (STI&hepatits) |
| **D:** ( TITLE-ABS-KEY ( "human immunodeficiency virus" OR aids OR hiv OR "acquired immunodeficiency syndrome" ) AND TITLE-ABS-KEY ( hepatitides OR hepatitis ) AND TITLE-ABS-KEY ( "sexually transmitted infections" OR std OR stds OR "sexually transmitted diseases" OR "venereal diseases" OR stis OR sti ) AND TITLE-ABS-KEY (integrat* OR pooling OR merge OR merging OR linkage OR combine OR join OR unified OR unify OR union OR consolidate OR incorporat* ) ) | (HIV&STI&hepatits) |
| **Search strategy of Web of Science =A OR B OR C OR D** | |
| **A:** ("Human Immunodeficiency Virus" OR AIDS OR HIV OR "Acquired immunodeficiency syndrome") AND ("sexually transmitted infections" OR STDs OR STD OR "sexually transmitted diseases" OR "Venereal Diseases" OR STIs OR STI) AND (integrat* OR pooling OR merge OR merging OR linkage OR combine OR join OR unified OR unify OR union OR consolidate OR incorporat*) | (HIV& STI) |
| **B:** ("Human Immunodeficiency Virus" OR AIDS OR HIV OR "Acquired immunodeficiency syndrome") AND (Hepatitides OR Hepatitis) AND (integrat* OR pooling OR merge OR merging OR linkage OR combine OR join OR unified OR unify OR union OR consolidate OR incorporat*) | (HIV &hepatits) |
| **C:** ("sexually transmitted infections" OR STDs OR STD OR "sexually transmitted diseases" OR "Venereal Diseases" OR STIs OR STI) AND (Hepatitides OR Hepatitis) AND (integrat* OR pooling OR merge OR merging OR linkage OR combine OR join OR unified OR unify OR union OR consolidate OR incorporat*) | (STI&hepatits) |
| **D:** ("Human Immunodeficiency Virus" OR AIDS OR HIV OR "Acquired immunodeficiency syndrome") AND ("sexually transmitted infections" OR STDs OR STD OR "sexually transmitted diseases" OR "Venereal Diseases" OR STIs OR STI) AND (Hepatitides OR Hepatitis) AND (integrat* OR pooling OR merge OR merging OR linkage OR combine OR join OR unified OR unify OR union OR consolidate OR incorporat*) | (HIV&STI&hepatits) |
