# Supplementary material for: Integration of HIV, Hepatitis B, and C, and sexually transmitted infections services: A scoping review of the benefits and challenges
Source: PLoS One. 2026 May 7;21(5):e0348073. doi: 10.1371/journal.pone.0348073 (PMC13152153; doi:10.1371/journal.pone.0348073)
Supplement: S2 Table — (DOCX) [file pone.0348073.s002.docx]

Table S2: Summary of characteristics of the documents included in the scoping review (n=118)

| Author(s) | year of publication | Country | Participants | Type of study | Aim | Integrated diseases | Benefits | Challenges |
| --- | --- | --- | --- | --- | --- | --- | --- | --- |
| Lei Zhang (10) | 2019 | Cambodia, | 370000 pregnant women | an economic evaluation | assess the population  impacts and cost-effectiveness of integrated approach in the Cambodian context | HIV, HBV and syphilis | 1.reduce the time required by both health care workers (19%) and pregnant women (32%) to further prevent mother to child transmission of these infections.  2.Estimated current mother-to-child transmission (MTCT) rates in Cambodia were 6.6% for HIV, 14.1% for HBV and  9.4% for syphilis. The integrated approach can significantly reduce HIV, HBV and syphilis MTCT to 6.1%, 3.4% and 4.6%, respectively 3. a net saving of $380,000 per year 4. highly cost effective ($64–$114 per disability-adjusted life years averted) |  |
| Jianhong Xia (95) | 2015 | China | 22 health agencies, 1600 pregnant women | a mix of qualitative and quantitative  methods with a needs assessment framework | assess the effectiveness of and examine challenges for integrated service delivery | HIV, syphilis, Hepatitis B | 1. facilitate the implementation of Prevention of Mother-To-Child Transmission of HIV | 1. poorly integrated service resources  2 interviewees ideas  may be only individual opinions rather than general  attitudes. 3.insufficient monitoring system to evaluate the effectiveness of integration of the services. 3. inadequate funding  4. the lack of integration of the information systems between different health agencies  5. Lack of a mechanism for coordinated management  6. Lack of the referral and communication networks with agreed guidelines and clear agency roles 7. Inconsistent outcome evaluation  8. no uniform guidelines, clear roles or consistent evaluation |
| Peter Whiticar (22) | 2007 | Florida and  Hawaii (US) | - | Perspectives | describe principles of integration,  identify potential benefits of and key barriers to integration, and recommend  changes to advance integration | HIV, STDs, and viral Hepatitis | 1. increase program efficiency in times of limited Resources by integration  2. Improving integration of program data systems  3. Increasing collaboration with immunization programs to  immunize at-risk adults  4. Increasing flexibility in using federal funding to support service integration  5. helps build new partnerships |  |
| Hillard Weinstock (91) | 2009 | Atlanta (US) | - | Viewpoint | highlights approach to the integrated use of data  by STD epidemiologists in the Outcome Assessment through Systems of Integrated  Surveillance (OASIS) workgroup. | STD, HIV, TB,  and Viral Hepatitis | 1.greater collaboration across disease programs 2.the ability to see problems and possible solutions in affected populations 3. sharing of technical expertise and innovative tools, |  |
| John W. Ward (114) | 2007 | - | - | Public health report | Integration of  HIV, STD, and Viral Hepatitis Prevention | HIV, STD, and Viral Hepatitis | 1.powerful approach to prevention  2. resulting in the release of STD treatment guidelines with a new emphasis on HIV infection and hepatitis B vaccination  3. the creation of STD/HIV prevention training centers |  |
| Ai-Ling Wang (11) | 2015 | China, | Pregnant women | Prevention program, longitudinal | China’s implementation of mother-to-child transmission for human  immunodeficiency virus, syphilis, and hepatitis B virus (integrated PMTCT services) | HIV,  syphilis and HBV | 1.feasible and  effective of Integrated prevention of three vertically transmitted  diseases –HIV, syphilis  and HBV at a large scale 2. offered all three tests concurrently, free of charge for pregnant women 3. Pregnant women infected  with HIV, syphilis or HBV were immediately enrolled for integrated PMTCT program services as a part of their routine antenatal, postnatal,  and children’s care. 4. Collaborated well by Maternal and child health clinics, the national and local Centers for Disease Control and Prevention and general hospitals and successful implementation of the program 5. helped China meet the WHO goal. | involved challenges including  shortage of human resources, quality  of services, and suboptimal availability  of services in some areas. |
| Julie Subiadur (32) | 2007 | US | people at high risk for infection | Longitudinal | describes the integration of viral hepatitis prevention services into the Public Health STD clinic, | Viral Hepatitis - STD | 1.Viral hepatitis services have been well received by clients and staff  2. viral hepatitis prevention services can be incorporated into a busy STD clinic, provided that leadership, staff, and resources are available | 1, Hepatitis A and B vaccination and HCV counseling, antibody testing, and referral services were reduced because of budget cuts. 2. most clients do not come in specifically for these services 3. in a busy clinic environment, hepatitis prevention services may not be prioritized when demand exceeds available resources  4. Full measurement of completion of the hepatitis B vaccine series was not possible due to inability to track individual client data |
| Suzan Stringari-Murray (12) | 2003 | Marin county, California | high-risk groups, 880 | Longitudinal, A program model | the results of the last 5 years of a program model of integrated services for HCV and HIV/AIDS, the challenges  involved, and the lessons learned | HIV-HCV | 1. proved a cost-effective way to implement HCV testing.  2. facilitated the referral of patients who needed transplant evaluation or were candidates for clinical trials 3. integration of health services conserves resources  4. Integration of testing services resulting in a 21% rate of positive HCV antibody tests.  5.The link between testing and the availability of a referral source for medical evaluation of clients who test positive | 1.the high comorbidity  of mental health and/or substance use issues,  2. demographic characteristics and medical complexity of patients presenting for evaluation, the  high rate of uninsured or underinsured patients, and  3.the lack of case management services for HCV.  4.The referral for patients with underlying diseases and additional evaluations delayed entry into treatment for HCV, and some patients were lost to follow-up. 5. yields a high prevalence of HCV-infected adults. |
| SHIELA M. STRAUSS (15) | 2005 | US | 89 Drug treatment units | Quantitative and qualitative study | examines the extent to which drug treatment units have expanded their HIV services to include those for HCV, and the extent to which this expansion was facilitated by having HIV services in place | HIV-HCV | provision of HIV training facilitated HCV staff training. | 1. having HIV education in place made it harder to implement HCV education for patients.  2.providing these HCV services for HIV may be insufficient to contain the epidemic among drug users. |
| Riley J. Steiner (62) | 2013 | US | - | Principles | Enhancing HIV/AIDS, Viral Hepatitis, Sexually  Transmitted Disease, and Tuberculosis Prevention in  the United States Through Program Collaboration and Service Integration | HIV/AIDS, Viral Hepatitis, Sexually  Transmitted Disease, and Tuberculosis | worth exploring to improve the effectiveness of program implementation and service delivery by providing more comprehensive prevention, treatment, and care to clients |  |
| Daniel Simoes (65) | 2021 | Local partners in Croatia, Italy, Lithuania and Poland (US) | 4 meeting, n=79 | methodology | the methodology to foster cross-disciplinary and cross-disease collaborations at national level as a vehicle for strengthened integration of testing and care services | HIV,  viral hepatitis, TB and STIs | implementation of national multi-stakeholder meetings as platforms for national level discussion |  |
| Daniel Simões (23) | 2022 | - | - | Perspectives | explore the current and future potential (as well as some concerns), importance, implications and necessary implementation steps for the use of platforms for multi-disease testing for TB, HIV, HCV, STI and potentially other infectious diseases, including emerging pathogens, using the example of the COVID- 19 pandemic | HIV, TB, viral hepatitis and STI | 1.cost sharing or joint procurement of equipment, reagents, consumables, staff, and service and maintenance across disease programs or even countries can leverage price reduction. 2. maximize contact with services by allowing for all relevant tests to be performed in as many sites as possible, providing a health response adjusted to the local epidemiology and the needs of populations served while simplifying patient pathways and possibly decreasing the number of visits and overall time for diagnosis of multiple infections 3. diagnostic integration would evolve to service and care integration for patients so they can access all necessary services across diseases through simplified and integrated service delivery |  |
| Robin Schaefer (102) | 2022 | - | - | Correspondence | HIV pre-exposure prophylaxis (PrEP) offers unique opportunities for integration with viral hepatitis services. | HIV PREP, Hepatitis | 1.PrEP scale-up could expand access to HBV care where access to services is suboptimal  2. Such integration can contribute to viral hepatitis elimination and generate health system efficiencies  3. tenofovir-based oral PrEP could be preferred for people with HBV because it suppresses HBV |  |
| Stephen Rudd (64) | 2013 | Pacific Northwest (US) | A clinic with a population catchment area of approximately 6500 tribal members | Letter to the Editor | HIV and chlamydia screening rates after integration | HIV, Chlamydia | The screening rate of chlamydia and HIV increased |  |
| Christina Rizk (53) | 2024 | New Haven, US | 173 HCV patients | retrospective | Describe monitor progress and provide flexible and innovative approaches  to facilitate engagement in HCV care, the  strengths of this approach and assessed for factors affecting the HCV treatment cascade. | HIV, HCV | 1. important to assess whether these efforts are scalable and constitute a sustainable approach for achieving WHO micro elimination targets.  2.Establishing a collocated HCV clinic within an HIV clinic model has been successful in facilitating pretreatment evaluation in 93.1% of coinfected patients with overall SVR12 documented in 56.1% of patients (79.5% of treated patients) | 1. lack of engagement in healthcare |
| Sarah Rhea (128) | 2018 | Durham County, US | 8431 patients were evaluated at the STD clinic and 733 unique, individual  Patients met the criteria for targeted HCV testing. Five of 7 STD clinic staff responded to the online  Survey. | Cross-sectional survey | Integrated Hepatitis C Testing and Linkage to Care at  a Local Health Department Sexually Transmitted  Disease Clinic: Determining Essential Resources  and Evaluating Outcomes | HCV, STD | 1. the HCV bridge counselor eased program implementation by providing HCV test results, education messages, and patient navigation services | 1.Replicability of this program to other region might also be limited by available funding  2. cost must be considered when planning integrated services  3. a dedicated HCV bridge counselor to provide test results, health education, and linkage-to-care assistance; and the establishment of local and accessible health care providers for HCV referral and management |
| D Raben (89) | 2020 | 20 countries in the conference from Europe and Romania | 65 abstracts | conference program | present the outcomes of the HepHIV 2019 conference, | Viral Hepatitis, HIV, STIs and TB | 1.the cost-effectiveness of frequent testing for risk groups,  2. response to the epidemics to better reach key populations | 1.criminalization of certain risk behaviors, 2. difficult to reach many of the people affected most by these diseases,  3.need political efforts |
| Thesla Palanee-Phillips (54) | 2023 | - | A total of 14 manuscripts | Editorial | Investigation of integration of HIV  prevention with sexual and  reproductive health services | HIV, sexual  services | 1.decreasing incidence of unplanned pregnancies, HIV and STIs.  2. improve equitable access,  3.yield holistic and comprehensive care,  4. raise the quality of maternal and antenatal care, 5.be cost-effective to the client and the health system, 6. increase financial sustainability with colocation of services and diversify healthcare provider capacity |  |
| Siobhan M. O’Connor (153) | 2019 | Guyana | 502 | Cross sectional survey | describes the first  effort to define the epidemiology of HBV and HCV infections and co-infections with HIV in  the Guyana general population or military forces, with associated risk factors, by integrating  HBV and HCV testing into HIV surveillance among military personnel | HIV, HCV, HBV | characterize HBV and HCV epidemiology, including probable recent transmission, prompting targeted responses to control ongoing HBV transmission, examination of hepatitis B vaccine policies |  |
| Van Thi Thuy Nguyen (30) | 2020 | Northern Vietnam | 2935 pregnant women | Pilot project | test the hypothesis that  combined universal screening for HIV, HBV, and syphilis  for pregnant women is feasible and contributes to  better health outcomes of exposed infants | HIV, HBV, and syphilis | 1.informed policy-makers of the feasibility of an integrated approach to prevention of mother-to-child transmission of HIV, HBV, and syphilis.  2. prevented the infant from contracting congenital syphilis from the mother by the timely treatment in early pregnancy.  3. 23 infections were averted by the pilot project’s additional interventions, including HBV testing for pregnant women, improved birth-dose coverage of HBV vaccine, and the provision of HBIG to infants with potential exposure during delivery |  |
| Joan Nankya‑Mutyoba (87) | 2022 | Uganda | 44 personnel from various hospital departments | pilot project | a pilot project to examine the feasibility and effectiveness of integrating care and treatment of HBV into routine HIV care services | HBV, HIV | 1.gain in HBV-related knowledge  2.a decline in stigmatizing attitudes  3. increase in mean scores  of total knowledge  2. Increase in mean correct knowledge scores across three knowledge domains (HBV epidemiology and transmission, natural history and treatment) post-intervention. | - |
| Thandar Su Naing (107) | 2023 | Myanmar | 715 HCV samples, seven laboratory staff involved in HCV, HIV VL, and EID testing | an integrated testing pilot | assessed the operational feasibility and acceptability of HCV/HIV integrated testing  implemented with a comprehensive package of supportive interventions | HCV, HIV | 1. useful,  2. its potential to expand testing  3.achieve cost savings | 1. fear of cross-contamination between HIV and HCV samples  2. Need staff |
| Joan Nankya Mutyoba (31) | 2023 | West Nile region, Uganda | 20 health care providers, 52 Patients | qualitative study | assess feasibility and acceptability of merging the care of HBV-monoinfected patients with existing HIV care system | HBV, HIV | 1.The integrated HIV/HBV care model is feasible and acceptable among both providers and recipients  2. a route to sustainable service delivery for HBV clients  3. an opportunity to leverage the existing HIV care system for HBV care | 1.Stigma arising from the Community  2. Stigma inherent within patient groups  3. reduced quality of services |
| Tanesha Moss (63) | 2014 | Miami, Florida (US) | 2,988 tests from clients of syphilis, hepatitis C, gonorrhea and chlamydia, and rapid HIV screening services | report | describe the prevalence of STIs, presents data on administering HIV and other infectious disease screening to individuals who might not otherwise be tested through traditional health services | Syphilis, Hepatitis C,  and Other STI, HIV | 1.an important means to identify infected persons, increase uptake of combination testing  2. minority, MSM, and transgender individuals who may be unable or reluctant to access routine screening in standard health-care settings can be screened |  |
| Yin Min Thaung (130) | 2017 | Myanmar | 803 HCV viremic (PLHIV, PWID, MSM, and FSW) | Longitudinal | 1.sustained virologic response (SVR) at 12 weeks after the end of treatment and cost per patient with SVR  2. safety (adverse events) during the treatment period | Hepatitis C, HIV and/or Hepatitis B virus-co-infected | increase sustained virologic response (SVR) rates for HCV among all populations, including PWID | 1.the estimated average cost of the intervention of $1250/patient is unaffordable for a national elimination strategy  2. cost of treatment, the cost and feasibility of HCV RNA testing, and significant virologic failure among PWID not on opioid substitution  therapy (OST) |
| R. Matulionytė1, (68) | 2021 | Lithuania, Romania and Spain | 3.664 consecutive individuals  aged 18–65 years participated: | Longitudinal | testing for  HIV, HCV and STIs. seborrheic  dermatitis, candidiasis, psoriasis, herpes zoster and  herpes simplex and STIs | HIV, HCV, HBV and STIs | Increased rate of test in all HIV, HCV, HBV and STIs  All HIV, HCV and HBV tests performed were antibody  tests and all individuals with a positive test were referred for further investigation and linked to treatment and care in an infectious disease hospital. |  |
| -Laferrière  Valérie Martel (118) | 2022 | Miami,  FL, and Montréal, Québec.  Canada | 500 persons  who injected drugs | multi-center, randomized, controlled, superiority trial | PrEP and HCV treatment  to those HCV-infected pwid | HIV, HCV | the individual benefits of HCV  treatment and the public health benefits of reduced on- ward transmission of HIV and HCV due to PrEP and HCV treatment, and cost-effectiveness of  offering PrEP and HCV treatment in healthcare venues frequently attended by PWID |  |
| Dianmin Kang (88) | 2013, | Shandong (China) | 3326 female sex workers | Post-intervention cross-sectional surveys | The integrated  individual level intervention, community mobilization and structural interventions included community solidarity and collective commitment, multisectoral government community partnership coordination, | HIV& STI | the significant lower rate of syphilis, higher score of HIV knowledge, and the  higher rates of condom use, HIV testing and the utilization of HIV prevention services in the intervention  sites, compared to that in the control sites, which suggested the successes of the integrated individual, community  and structural intervention. This  study demonstrated that the integrated individual, community, and structural intervention have positive impact  in reducing HIV and STI risks among FSWs. | Structural factors including stigma and discrimination are critical concerns  among this FSW population and needed to be  addressed to enable vulnerable women to adopt |
| Heidi L. Hoffman, (55) | 2004 | Massachusetts |  | Report (expertance) | HIV, Hepatitis,  Addiction Services Integration | HIV& Hepatitis | Integrated approaches can promote greater efficiency, improving  communication and coordination among clients, providers, and government  funding agencies. saved costs. Include more comprehensive range of Service. Such systems would enable providers to be more responsive to clients with  multiple and complex needs, and would likely result in better access to a more comprehensive range of services for these clients and others at risk. | one challenge has been the need for providers to communicate with multiple bureaus within MDPH in order to access state funds to support their efforts. absence  of an established integration framework, providers chal-  lenged MDPH to provide guidance. Collaborative planning was needed. |
| Robin R. Hennessy (59) | 2007 | New York City (US) | 1000 people such as IDUs and others | Longitudinal | integrated service delivery to high-risk IDUs | HIV& hepatitis& STIs | clients coming to the clinic for hepatitis vaccine or screening only cheeked for HIV and STDs. many also benefited from STD/HIV exams, testing, treatment, and referrals they may not have received otherwise | concerned about overburdening staff. Clients  are likely to underreport injection drug use, which may underestimate the impact of integrated services  on IDUs. the client refused additional  recommended services (HCV and HIV testing), |
| ROBERT A. GUNN,(120) | 2000 | San Diego, California (US) | 350 clients such as IDUs and MSM | Longitudinal | Screening for Chronic Hepatitis B and C Virus Infections in STI clinic | HIV& Hepatitis& STIs | Integrating hepatitis services into an STD clinic and field investigation program should better serve the needs of high-risk clients, such as MSM and IDUs, and provide an opportunity for STD, HIV, and hepatitis prevention and control services to be delivered in an efficient and effective manner |  |
| LISA K. GILBERT (97) | 2005 | US | STD program managers and STD clinic managers | Report | assess improvements since 1997 in hepatitis B prevention integration in STD services. | HBV and STD | Large increases were found in the percentage of clinics offering hepatitis B vaccine (from 61% to 82%), providing education (49% to 84%), and accessing federal vaccine programs (48% to 84%). Twice as many program managers considered all patients with STDs eligible for hepatitis B vaccination. | lack of  funding, lack of resources to track patients, and client (non)compliance  with vaccine series completion. |
| Kevin A. Fenton (46) | 2014 | United  States |  | Guest Editorial | prevention  and control of human immunodeficiency virus  (HIV) infection, viral hepatitis, sexually transmitted  diseases (STDs), and tuberculosis (TB) | HIVS& STIDs&  TB | interrelated prevention strategies in an effort to provide more comprehensive  delivery of services, support improvement in the efficiency of service  delivery, increase programmatic cost-effectiveness, and improve health outcomes. costs were low. referrals for outside care. benefits to  integrated testing. people who were  previously diagnosed with HIV but fell out of HIV care were reengaged in care |  |
| David Livingstone Ejalu (110) | 2022 | West Nile region of  Uganda (Arua &Koboko) | Data were extracted from 3121 files of HIV and  hepatitis B virus (HBV) monoinfected patients from the two  study sites. | cross-sectional  hospital-based  cost  minimisation study | To estimate provider costs associated with  running an integrated HBV and HIV clinical pathway for  patients on lifelong treatment | HIV&HBV | Findings showed that Arua hospital had a higher cost per patient in both clinics than did Koboko Hospital. The cost per HBV patient was US$163.59 in Arua and US$145.76 in Koboko while the cost per HIV patient was US$176.52 in Arua and US$173.23 in Koboko. The integration resulted in a total saving of US$36.73 per patient per year in Arua RRH and US$17.5 in Koboko DH. |  |
| Peder Digre (36) | 2021 | Jackson, Mississippi | with 8 DIS and case tracking forms for 90 unique cases | qualitative | Integrating HIV-Related  Activities Into Sexually Transmitted Disease Partner  Services | HIV& STIs | was generally low cost and acceptable  to the disease intervention specialists (DIS).  but initiating new work (STD partner services  for HIV-negative MSM with gonorrhea/ chlamydia) was relatively  costly. | disparate data systems, nonsystematic documentation,  and lack of training as barriers. Noted that it was an increase in their workload. Environmental barriers  such as stigma and distrust of the health department limit the ability of the DIS to conduct outreach to index cases and their partners. |
| Catelyn Coyle (66) | 2016 | Philadelphia, Pennsylvania (US) | four community health centers | Before-after  Interventional | HCV/HIV Testing &Linkage to Care | HCV&HIV | Increased HCV and HIV screening (increased testing HCV and HIV) enhanced seropositivity diagnosis, and improved linkage to care. |  |
| Kipruto Chesang (27) | 2017 | Kenya | 87 healthcare providers | qualitative  thematic analysis | managing sexually transmitted infections in  HIV care settings | STIs& HIV | STIs facilitated HIV transmission and that by controlling STIs, HIV would also be controlled. The advantages of integrating STI and HIV services mentioned were stigma reduction (particularly among men), saving time, improving patient management through capturing better quality sexual history, early detection of STIs and better contact tracing, improving confidentiality  and healthcare provider /patient relationship, easing access by providing a one-stop service, and providing  a holistic package of services. Providers also said that integration supports the health system by enabling availability of appropriate equipment for screening, reducing missed opportunities for treating STIs, being less costly overall due to shared resources, having fewer STI drug stock-outs through integrated management, and improving reporting. | low commitment by higher levels of management, few recent STI-focused trainings,  high stigma (some  healthcare providers thought integration would increase stigma as some patients with STIs might not want to be associated with HIV through STI services being co-located with HIV services) and low community participation, and STI drug stock-outs.  the shifting national policies on integration, the conflict of  charging for STI services while HIV services are free, heavy workload, inadequate knowledge  on STI management, and high staff turnover |
| Tembe Carveth-Johnson (67) | 2021 | Gaborone, Botswana | 451 patients | prospective study | Integrating Sexually Transmitted Infection Testing  and Treatment with Routine HIV Care | STIs& HIV | integrating STI testing into routine  HIV care was feasible. All participants were able to provide adequate self-collected samples, were informed of their results, and were provided with treatment if required. tested positive were given treatment and counseled regarding partner notification. Pregnant women were invited for a test of cure after 4 weeks. |  |
| Doug Campos-Outcalt (37) | 2006 | Maricopa County (US) | 17875 patients | Longitudinal study (18 months) | Integrating Routine HIV Testing  into a Public Health STD Clinic | STIs& HIV | HIV testing can be included in the routine battery of tests offered  at an STD clinic with high patient acceptance. Routine testing can discover  those who are unaware of their HIV-positive status, providing an opportunity  for early referral for treatment, counseling to avoid disease transmission, and notification of sexual contacts. |  |
| Julie Bottero (28) | 2016 | France | group of 19 ‘testing experts’ | guideline | define a comprehensive testing strategy for chronic viral infections, emphasizing both targeted screening and mass screening and considering jointly HBV, HCV and HIV. | HBV, HCV, HIV | Combine the testing for the chronic viruses (namely, HBV, HCV and HIV), given the epidemiological  similarities, and in order to simplify screening indications and favour empowerment by all medical and associative. The development of HBV and HCV POC tests enables community initiatives to propose screening interventions for these infections, along with HIV. As for the POC HIV tests used for many years, these tests require minimal equipment and a puncture capillary whole blood from the fingertip or a simple crevicular liquid sampling. Easy to perform, including by nonmedical personnel, the result is obtained in less than 30 min. | Some barriers to screening implementation are related to the widespread lack of knowledge about viral hepatitis and their exposure to risk factors among the general population. The main barriers to testing are the physician unawareness of the patient exposure to a risk situation and his/her ignorance of some risk factors |
| Tanisha Bharara (116) | 2019 | India | A total of 44738 ICTC attendees were tested for HIV | retrospective study | INTEGRATION OF HIV TESTING WITH TUBERCULOSIS AND SEXUALLY TRANSMITTED INFECTIONS | HIV-TB and HIV-STI | For HIV positive (approximately 3%).  Seropositivity was found to be highest in the reproductive age group (25–49 years). A decline in HIV seropositivity rate was seen, though the number of clients increased by approximately 20% each year. HIV-TB co-infection rate was found to be 0.18% in 2016, 0.15% in 2017 and 0.17% in 2018 among patients attending DOTS (Directly Observed Treatment Short-Course) centre. HIV positivity among patients referred from STI clinic showed a declining trend from 2.5% in 2016 to 1.35% in 2017 and 0.6% in 2018. |  |
| Tigran Avoundjian (93) | 2019 | Mississippi (US) |  |  | improve HIV case finding | HIV and syphilis | opportunities for both syphilis and HIV prevention, and integrating HIV prevention activities, such as HIV testing, into syphilis partner services in Mississippi could have a positive impact on HIV prevention efforts. Integrating HIV testing into syphilis partner services is potentially an effective strategy for identifying new HIV positive cases in Mississippi, particularly among partners of Black/African American MSM and previous HIV positive index cases. Given the high-test positivity among these subgroups, increasing the proportion of HIV negative partners tested for HIV could potentially increase HIV case finding through syphilis partner services. we were able to identify the proportion of HIV-negative partners that were not tested because they were unable to locate, refused examination, or refused partner services | the reasons for a significant proportion of HIV-negative partners that were not tested remain unclear. HIV testing, in particular negative HIV test results, are not routinely documented in the MSDH STD surveillance data system. |
| Noelle Cocoros (33) | 2014 | Massachusetts, US | 2,716 inmates/detainees | a pilot a pilot longitudinalprogram | assess the feasibility of integrating HCV screening into an HIV screening program in a correctional setting | HCV, HIV | 1. feasibility of integration of HCV education and screening into correctional facilities,  2. increased opportunities for education, testing, and linkage to care | 1.Need high level of cost for effective linkage to care and. costs associated with HCV care and treatment  2. difficult to implement in other states and facilities |
| Kevin A. (38) | 2013 | London (UK) | - | program | to develop and implement a more syndemic approach to prevention. / appropriateness, feasibility, effectiveness, acceptability, and accountability of prevention programs for HIV, STDs, viral hepatitis, and TB infections by leveraging new opportunities and reducing missed opportunities for collaboration and integration | HIV, STDs, viral Hepatitis, and TB | 1.training of project officers, and leadership to promote partner awareness and acceptance  2. complements and enhances disease-specific programs and services | 1. narrow funding streams,  2. not conducive of leadership and management cultures to integration, 3. inadequate payment and performance incentive schemes  3. need leadership engagement and organization support  4. staff training  5. support and use of technology |
| Vicky Bungay, (14) | 2013 | Canada, China, European countries, US | 129, Community sex workers | A model approach project, longitudinal | the design and implementation of a CHW Model HIV Prevention and Health Promotion Program that expanded the health education, primary care referrals, and health appointment accompaniment services to include STI testing using self-swab techniques and a point-of-care (POC) HIV screening test | HIV, STI | 1. facilitated the development of trusting and supportive relationships with participants by repeat visits  2. the convenience of onsite and rapid testing methods  3. contributed to the acceptability of HIV and self-obtained STI tests  4. increase health care access and testing uptake  5. providing health education, and improving women’s access to other health services | 1. offering limited testing services  2. lack of awareness of sexual health clinics, 3. language barriers, knowledge deficits |
| Neela D. Goswami (69) | 2012 | North Carolina (UK) | 247 community and clinic population | cross-sectional | feasibility and case detection rate of a geographic information systems (GIS)-based integrated  community screening strategy for tuberculosis, syphilis, and human immunodeficiency virus (HIV). | Tuberculosis, HIV, and syphilis | 1. increase the impact of screening  programs organized by resource-limited health departments  2. important for efficiency,  3.to detect co-infection  3. takes advantage of shared risk factors, and targets persons with multiple infections | 1. Continued innovation is needed to improve  2. facilitate deeper penetration into the highest-risk populations  3. linkage to care must be improved for being a cost-effective  4. financial incentives |
| Nicole HTM Dukers-Muijrers (47) | 2012 | Netherlands | 447 patients receiving HIV care | Pre- and post-test intervention | close the gap in sexual health care  by implementing and evaluating a policy change regarding  the combination of public health care and hospital care in  an innovative, integrated STI /HIV care structure | HIV, STI | one-quarter of HIV patients used the integrated sexual healthcare services  3. patients were satisfied with the care offered  4. diagnosed a substantial number of asymptomatic, mostly anorectal and oropharyngeal, STIs in MSM  5. facilitates multidisciplinary work and contributes to sustainable partnerships, good rated for quality of care | 1. need coordination of individual-level hospital and public health services  2. requires increased awareness among patients  3. need cultural shift in hospital and public health organizations |
| Joseph D Tucker (94) | 2010 | Hong Kong | personals (clinicians, laboratory technicians, and nurses) of eight STI clinics | A qualitative study | analyzed the clinical, laboratory, and human capacity at various types of STI clinics in China to inform implementation of integrated syphilis/HIV testing efforts and guide scale-up policy | Syphilis/HIV | - | 1. need economic resources  2. need accurately recording physician reported information about reimbursement  3. financial incentives |
| Kathleen A. (48) | 2005 | US | providers who treat HIV-infected patients and gastrointestinal or liver specialists, patients | Program | a program to shift the primary responsibility for oversight of care for HCV-infected patients from the liver clinic to HIV primary care clinicians and to provide education and support regarding adherence to patients | HCV, HIV | Improving outcomes of treatment among coinfected patients | 1.Communication between primary care and liver clinics was paper based, slow, and too unreliable to allow for coordination and monitoring of a complex treatment regimen |
| Czarina N (129) | 2021 | US | Fifteen leadership and staff from seven MMT programs with on-site HIV/HCV testing | Qualitative study, survey | understand how methadone maintenance treatment (MMT) programs that offer on-site HIV and/or HCV (HIV/HCV) testing have integrated testing services, and the challenges related to offering on-site HIV/HCV testing | HIV, HCV | - | 1.the absence of state policies that facilitate medical billing and inconsistent grant funding  2. Testing availability was limited  3. Need technical assistance  4. need policy changes related to privacy  5. lack of effective approaches for integrating addiction health services and medical care |
| Janneke P Bil (39) | 2018 | Amsterdam Gelderland (Netherland), | 859 eligible migrants | Longitudinal (a screening project) | evaluate whether integrated TB, HBV, HCV and HIV screening is effective and acceptable among migrants | HBV, HCV and HIV, TB | About half of the migrants visiting the five TB departments accepted HBV, HCV and HIV screening, | 1.more data and cost-effectiveness studies are needed for decision-making  2. not all migrants are registered with a GP or have a Dutch health insurance at the time of screening |
| Julie Bottero (70) | 2015 | France | 327 representing mainly African immigrants | Randomized Control Trial | evaluating the  feasibility of an intervention based on simultaneous HBV, HCV,  and HIV tests as a means to promote screening and linkage-  to-care in a population without healthcare coverage, almost  exclusively immigrants, with high risk of viral infection | HBV, HCV,  and HIV | 1. improve the overall cascade of screening and consequently linkage-to-care in a population with high risk of chronic viral infection | 1. individual’s motivation to seek an appropriate center |
| Edward R. Cachay (13) | 2021 | Spain, US, california | Fifty-four consecutive persons with HIV co-infected with hepatitis C | retrospective cohort analysis | Hepatitis C Treatment Outcomes  in Persons With HIV and Decompensated  Cirrhosis Using a Collaborative  Multidisciplinary HIV-Centered Approach | HIV, HCV | 1.. success in developing effective cures for HCV requires us to find ways to treat HIV  2. Possibility of HCV treatment success | 1.need foster inclusion and collaboration among multiple providers caring |
| JOAN M (57) | 2009 | - | Previous studies | Editorial | Responding to the Burden of STD, HIV, and Viral Hepatitis in  Correctional Populations through Program Collaboration and Integration | STD, HIV, and Viral Hepatitis | 1.provide better and more efficient care for the most vulnerable  2. a key strategy for tackling disease syndemics | require programs to demonstrate a willingness to encourage cross-agency thinking, risk taking, and doing work that is beyond the experience, mission, and task of any single agency |
| Ethan Cowan (80) | 2018 | US | 478 adult patients | two-armed, randomized controlled trial | determine the effect of integrating rapid HCV testing into an established HIV testing program | HCV, HIV | 1.may improve population health by identifying and linking infected individuals to care  2 an effective and efficient approach to screen at-risk populations | - |
| Helen Eborall (40) | 2020 | United Kingdom | 9 focus group with migrants (74),  semi-structured interviews with health care professionals (32),  individuals having tested positive for one/more infections (23) | Prospective qualitative study | Understand the views of migrants and health care professionals to combine screening for multiple key infectious diseases including latent TB, HIV, hepatitis Band hepatitis C | TB, HBV, HCV, HIV | 1. well-received screening program by migrants and professionals, feasible and acceptable | 1. problem with data sharing |
| Lauren F. (98) | 2014 | - | in 59 CDC-funded  health department | Annual progress reports | identified the level and type of program collaboration and  service integration (PCSI) among HIV prevention programs in 59 CDC-funded  health department jurisdictions. | HIV/STD, viral hepatitis, and TB | 1. Integrated testing, education and  outreach activities and partner services as the main service integration  activities. | 1. State health departments differ structurally and fiscally and have distinct epidemics to address |
| H.DawnFukuda  (24) | 2020 | US, Massachusetts | Data system | Case study | define policies that contributed to successful integration | HIV, STI, viral Hepatitis, and TB | 1.improvements to laboratory, surveillance, testing, and linkage-to-care capacities relative to HIV, HCV, STI, and TB 2. increased HIV and HCV tests and new HCV diagnosed  3. prompt identification of outbreaks  4. rapidly deploy interventions | 1.took substantial time and resources  2. need investment in training, education, and development of system capacity |
| Robert A. (121) | 2005 | California | 930 clients | Longitudinal | an evaluation of the integration of  prevention services in an alternative sentencing drug rehabilitation program | HIV, HBV, HCV, STD | 1. help people at increased risk of HIV, hepatitis, STD  2. high rates of success in providing hepatitis prevention services and expand hepatitis services | 1. need cost management |
| ROBERT A. (41) | 2007 | San Diego, California | 21,631 clients of STD clinic | Longitudinal | evaluation of a hepatitis B vaccination program among STD clinic clients | HBV, STD | 1.Although the lower vaccine coverage rates from childhood and adolescent, it could be acceptable for the population of STD clinics  2. comparable to the vaccine coverage rates in other adult populations  3. vaccine acceptance (69%) and completion rates (second dose, 55%, and third dose, 33%), higher than previously reported acceptance rates (50%–44%)  4. increase in vaccination coverage over time | 1. cost of hepatitis B vaccine and need fpr incorporate funding for vaccine |
| LISA HANSEN (96) | 2005 | British Columbia | 1200 primary care physicians (407 response) | Longitudinal | evaluated British Columbian primary care physicians’ use of sexually transmitted disease (STD) and HIV counseling guidelines, assess barriers to integrated HIV and STD testing and counseling | HIV, STD | 1. two thirds of all physicians, and significantly more female physicians than their male peers, always offered HIV testing to anyone requesting it | 1.need accessible and relevant continuing medical education in novel formats  2. lacked sufficient information on HIV and STD risk and prevention by physicians in nonurban areas  3. guidelines are theoretically equally available to all physicians, but they are not universally used  4. limited continuing education opportunities  5. less confidence in ability by nonurban physicians |
| Jennie L. (131) | 2007 | United States | six STD clinics (client visits ranged from 2,883 to 23,109 per year) | a retrospective study | examines trends in hepatitis B vaccination  from 1997 to 2005  that were committed to integrating  viral hepatitis prevention services—including hepatitis  A and B vaccination and hepatitis C virus  counseling, testing, and referral—with existing clinical  services. | HBV, STD | 1.Increased rate of hepatitis B vaccination in the STD clinic by 54% from 2002 to 2003  2. STD clinics can implement hepatitis B vaccination and reach large numbers of high-risk adults | 1.need adequate funding and vaccine supply |
| Gary Heseltine (104) | 2007 | Texas, US | counselors (13) and managers (13), clients of health department | Program report | a statewide hepatitis C education and prevention program and incorporating into training for all HIV, STD, and substance abuse counselors. | HCV, HIV, STD | 1. the most efficient way to reach people at risk of or infected with HCV | 1. handle the increased workload  2. The lack of referral resources |
| A. Jewett (25) | 2013 | US | STD clinic clients (interview with 3 HCV positive, 9 HCV negative, Focus groups with triage staff, providers, and linkage-to-care counselors. question for 926 clients who reported at least one risk factor, 876 client testing) | Longitudinal, qualitative assessment | facilitators and barriers to integrating HCV point-of-care testing into standard operations at an urban STD clinic | HCV, STD | 1.pleased with the ease of use and rapid return of test results  2. increase identification of persons with HCV infection  2. an opportunity to increase awareness | 1. loss to follow-up and access to care  2. questions of clients that fell outside the scope of testing  3.need involving all stakeholders in the development and implementation of the project  4. need resources |
| Jack Jourden (71) | 2004 | US | - | Viewpoint | the programmatic split between prevention efforts as well as an overview of attempts to call attention to the need for enhanced program consolidation related to STD and HIV | HIV, STD | 1.Increased flexibility in offering STD screening alongside HIV testing and treatment  2. Better cross training and utilization of staff and administrative time | 1. Increase funding  2. The different nature of AIDS and other sexually transmitted diseases  3.stigma about HIV  4. Competition for funding between programs |
| David A. Katz (115) | 2018 | King county, Washington (US) | 7546 cases of early syphilis, gonorrhea, or chlamydial infection among HIV-negative MSM | longitudinal | evaluated the integration of pre-exposure prophylaxis (PrEP)  referrals into STD partner services (PS) for MSM | HIV (PREP), STD | 1.feasible and effective at increasing PrEP uptake, particularly among men at highest risk of HIV  2. an opportunity to address barriers to PrEP use and offer additional referrals  3. 1 of every 10 MSM receiving STD partner services can successfully referred to initiate PrEP | - |
| XMirjam-ColetteKempf (42) | 2018 | Alabama and Mississippi (US) | 8 to 12 participants for each 6-focus group, community stakeholders (31) | Longitudinal, qualitative & quantitative study | evaluate the acceptability, feasibility, and best practices of an integrated HIV and hepatitis C virus community-based health screening approach | HIV, HCV | 1.acceptable and feasible;  2. overcome existing barriers of stigma and discrimination | 1. need training and education based on culture |
| Thomas F. Kresina (103) | 2015 | - | - | Supplement article | Integrating Care for Hepatitis C Virus  and Primary Care for HIV for Injection Drug Users  Coinfected with HIV and HCV | HIV, HCV | 1.a trusting relationship with their HIV care provider for facilitates decisions about beginning HCV treatment  2. clinicians treat HCV infection in their patients rather than referring within the clinic to other specialists | 1. need to trained HIV specialists and remain current on treatment for HCV infection |
| Jean De Dieu Longo (26) | 2018 | Africa | 279 patients | Cross sectional | simultaneous detection of HIV and HCV and HBV in STI clinic | HIV, HBV and HCV. | 1.strengthen the prevention and therapeutic strategies already in place  2. more advantageous by maximizing available resources  3. providing rapid results 4. improve the screening of highly prevalent viral infections 5. reduced cost. | - |
| Ralph-Sydney Mboumba Bouassa (85) | 2018 | Africa | 266 child bearing women | Brief report | simultaneous detection of HIV and HCV and HBV | HIV and HCV and HBV | 1. improve all at once the “cascade of screening,” prevention strategies, and linkage to care with reduced cost | - |
| Kwame Owusu-Edusei (106) | 2014 | China | 10,000 pregnant women | Cost effectiveness study | assess the health and economic outcomes of different  strategies of prenatal HIV and syphilis screening from the local health  department’s perspective | HIV, Syphilis | 1.if an existing HIV- strategy added syphilis screening was $140 per additional DALY prevented,  2. HIV screening programs with syphilis screening are more cost-effective than HIV screening alone  3. prevent many more adverse pregnancy outcomes |  |
| Abdulmumin Saad (99) | 2012 | Nigeria | 240 undergraduate students | A randomized controlled | evaluate the effectiveness of a peer led  human immunodeficiency virus-sexually transmitted  infections (HIV-STI) intervention program based on the  Information-Motivation-Behavioral Skills model | HIV and STI | 1.a salutary impact on knowledge, behaviors, and attitudes related to HIV and STI  2. improving HIV and STI knowledge, sexual risk behavior, and attitudes toward prevention of HIV | - |
| Joseph D. (81) | 2011 | China | 2061 STI patients in 6 STI clinics | Pilot study | use multilevel modeling to  analyze determinants of syphilis and HIV-testing uptake at STI clinics  in China, Integrated HIV/  Syphilis Testing at STI Clinics | HIV, syphilis | 1.The high level of integrated syphilis/HIV test uptake, 81%, found improvement compared with alone HIV testing.  2. the high level of integrated syphilis/HIV test acceptance | - |
| Bao Ngoc Vu(44) | 2022 | Vietnam | 8840 key pop | Cross sectional Project | Effectiveness of community-based  and  facility-based  HBV/HCV screening | HIV, HBV, HCV | 1.high acceptability among key pop  2. a powerful tool for increasing HBV/ HCV uptake  3. showing which KP may be at highest risk of HBV/HCV infection in particular settings  4. access free ART or PrEP using a tenofovir regimen that is also effective for HBV | 1.need for efforts to ensure low cost or free treatment |
| Baoxi Wang (122) | 2015 | China | 8275 women | Longitudinal | the effect of an integrated  comprehensive structural STI/HIV intervention on incident  syphilis and HIV infection (screening) | STI, HIV | 1.reduce syphilis incidence among FSWs  2. focused on creating mutual trust and sustainable connections between sex venues and STI clinics  3. May provided greater leverage for FSWs to mandate condom use | 1. feasibility and the cost-effectiveness require further investigation |
| Evy Yunihastuti (49) | 2021 | Indonesia | 2094 HIV-infected patients | a retrospective cohort | describe the HCV  continuum of care of HIV-infected patients treated in an HIV clinic after a free DAA program | HIV, HCV | 1.facilitated a better HCV care  2.high effectiveness | - |
| Richard Zimmerman (92) | 2007 | US, Illinois | STD clinics clints | Program | Integrating Viral Hepatitis Prevention  into STD Clinics | Viral Hepatitis, STD | 1.a cost-effective public health strategy when long-term consequences of these diseases are considered  2.contribute to expanding viral hepatitis prevention | 1.need a statewide data system  2. inadequate resources available  3. limited funding |
| Janet Wilson (50) | 2019 |  |  | Report of conference | integration | STI&HBV&HIV | integration of STI services (including  hepatitis B and HPV vaccination), reproductive health services (including contraception and cervical cancer screening), and HIV treatment and care. Partner notification and treatment would be a core component of the services. infant vaccination at birth) with combined efforts to reduce mother to child transmission of HIV, syphilis and hepatitis B. also include advice about protection against STIs, HIV and unplanned pregnancies and initiatives to promote behaviour change, with the provision of male circumcision, pre-exposure prophylaxis and post-exposure prophylaxis for the prevention of HIV. There would be assessments for drug and alcohol use/addiction and provision of, or referral to, services to reduce these |  |
| Natalia Villegas, (100) | 2015 | Santiago, Chile | Forty young Chilean women between  18 and 24 years of age | prospective cohort study (pretest–  intervention–posttest) | STI and HIV  prevention-related information, motivation, behavioral  skills, risk behaviors, and IPV. | STI and HIV | women reported a significant increase in levels of STI- and HIV-related knowledge, attitudes toward the use of condoms and perceived self-efficacy, and a reduction of risky sexual behaviors with uncommitted partners. |  |
| Isidore T Traore (123) | 2015 | Burkina Faso | 321 HIV-uninfected FSW | prospective, interventional cohort study | The intervention combined prevention  and care within the same setting, consisting of peer-led education sessions, psychological support, sexually transmitted  infections and HIV care, general routine health care and reproductive health services | HIV & STI | No participant  seroconverted for HIV during the study.  This null incidence was related to a  reduction in the number of regular partners and regular clients, and by an increase in consistent condom use with casual clients  and with regular clients |  |
| Thomas J. Stopka (72) | 2007 | California | injection drug users (IDUs) | Intervention study | HCV and HIV Counseling and  Testing Integration | HIV & HCV | Increasing HIV counseling and testing (C&T) rates and test results disclosure rates. Capitalizing on existing disease screening centers, trained field staff, and public health infrastructures allows for HIV and HCV C&T integration to take place without substantial budgetary increases and with minimal extra training required | Integrating HIV and HCV C&T increased overall C&T time required for staff and clients and increased stress among counselors due to the number of positive test results (HCV) given to clients. |
| Laura E. Starbird (56) | 2019 | Baltimore, Maryland, USA | individuals age 18 years or older with HIV and chronic HCV | a randomized controlled trial comparing | 1. included a nurse-initiated HCV referral, strengths-based HCV education, patient navigation and clinical coordination, and appointment reminders.  The goal of Phase 2 was to minimize potential ART/DAA drug interactions to reduce time to HCV treatment initiation. | HCV and HIV | case management can improve linkage to HCV care among vulnerable persons co-infected with HIV by coordinating specialty referrals, navigating appointment scheduling, providing strengths-based education and tailoring appointment reminders. | without a care delivery system that promotes HCV treatment in this high-priority patient population, such as evidence-based treatment decisions and HIV providers trained and willing to treat HCV, the benefits of case management may be limited. |
| Sunil Suhas Solomon (79) | 2019 | India | People who inject drugs (PWID) | A cluster-randomized trial | Integrating | HIV and HCV | significant impact of integrating HCV testing with HIV/harm reduction  services on critical HCV care continuum outcomes including HCV testing, awareness of status and treatment initiation. |  |
| A Smith (76) | 2013 | rural Guatemala | pregnant women | a simultaneous triple point-of-care screening program | aim was to evaluate its feasibility and effectiveness in increasing testing uptake, case detection and referrals for positive cases. | Syphilis, Hepatitis B & HIV | 65% were screened for HIV and syphilis and 62% for HBV. Testing uptake increased 209%/30% from baseline for HIV/syphilis (p < 0.001). |  |
| SeyedAhmad SeyedAlinaghi (52) | 2019 | Iran |  | a purposive sampling method |  | HIV, Hepatitis C and Tuberculosis | it seems that these settings are a good opportunity to introduce the protocol for early detection of people living with HIV to achieve higher coverage of HIV care and treatment services opportunity for improved prevention and management intervention programs, enabling clients at risk or those who have been diagnosed with a target disease to be followed up and receive the appropriate health care. the overall wellbeing and quality of life among these groups may be significantly improved. Ultimately, such practical protocols will help to reduce morbidity and mortality among clients with HIV/HCV/TB and the broader community. |  |
| Bruce R. Schackman (117) | 2007 | Haiti | pregnant women | A decision analytic model simulated | Cost-Effectiveness of Rapid Syphilis Screening in Prenatal HIV Testing Programs | Syphilis and HIV | integrating a new rapid syphilis test into prenatal care and HIV testing would prevent congenital syphilis cases and still births, and is cost-effective. |  |
| G S_anchez (109) | 2016 | Mexico |  | modeling | The cost-effectiveness | HIV, tuberculosis and hepatitis C virus | it is possible that the population with triple infections could achieve important benefits in terms of years of life gained. | It is known that treating patients with HIV who are coinfected with TB or HCV implies high cost and low efficacy |
| William S. Pearson (124) | 2018 | the United States | American patients and providers use self-tests |  | Would American patients and providers use self-tests for gonorrhea and chlamydia | gonorrhea and chlamydia | Among our sampled population of men who have sex with men, 79.5% said they would prefer to take this type of test at home and 73.9% said they would be willing to pay at least $20 for the test. Among young adults (18–29 years), 54.1% indicated that they would like to take this test at home and 64.5% were willing to pay more than $10 for such a test. Among sampled physicians, 85.1% were “likely” or “very likely” to use an FDA-approved STD self-test in their office to screen for Chlamydia trachomatis (CT) and Neisseria gonorrhea (GC) |  |
| Jason J. Ong (77) | 2022 | Australia | 33 semi-structured individual interviews | Hub-and-Spoke Model: A Mixed-Methods Evaluation | Improving Access to Sexual Health Services in General Practice | HIV and STI (chlamydia, gonorrhoea, syphilis) | There was a statistically significant rise in testing for HIV and STIs in all general practices. increase of an average of 11.2 chlamydia tests per month 10.5 gonorrhoea tests  4.3 syphilis tests  5.6 HIV tests |  |
| Kyegombe NambusiI (78) | 2023 | Gaborone, Botswana | 200 young people (female and male) aged 18–24 years | intervention | evaluating dual self-testing for HIV and STIs among young people in Gaborone. | HIV, Hepatitis and Syphilis | that most of the young people were already aware of their HIV status and were motivated to participate in self-testing mainly because they were interested in learning their STI status. | Both young people and healthcare workers raised concerns about the potential negative mental health outcomes of unexpected test results and emphasized the importance of pre- and post-test counselling and seamless linkage to care. |
| Roman V. Mednikov (16) | 2016 | Russia | nine patients co-infected with  HIV and HCV genotype | intervention | Combining drug therapy | HIV and HCV | The results of our study have shown that the positive clinical effect in the treatment of HCV patients by  DFPP (double filtration plasmapheresis) is also achieved in the treatment of HIV infected patients. A conclusion can be made that the complex therapy of hepatitis C, including DFPP and  medication by PEG-IFN + RBV is an effective and safe approach for the treatment of HCV in patients  co-infected with HCV and HIV. |  |
| Javier Martínez-Sanz (73) | 2020 | Spain | 7,991 participants | a cluster randomized trial | evaluate the effect of a combined screening program | HIV and HCV | The overall screening coverage was higher within the intervention arm (OR 17.7; 95% CI 16.2–19.5; p<0.001).  The rate of HCV diagnoses was higher among intervention centers |  |
| VD Mandel (74) | 2018 | Italy | collected 13117 admittances for 9154 patients | intervention | The main objective was to analyze the number of screening tests performed in the T&C-IDC and STDs-DU, comparing the results obtained after the adoption of the shared protocol with the previous period. The secondary aim was to evaluate the linkage to care of newly diagnosed patients. | HIV, Hepatitis and syphilis | Increase screening tests and ratio between tests and admissions.  the most common was syphilis (41.9%), followed by HBV (25.7%), HCV (21.4%) and HIV (10.9%). |  |
| J. Leenen (43) | 2020 | Netherlands | HIV positive patients. | Intervention | determine the factors for the successful implementation of STI home sampling strategies. | chlamydia, gonorrhoea, Hepatitis B, and syphilis  HIV | The home sampling programme increased STI test uptake and was acceptable and feasible for MSM and their care providers. |  |
| David W. Hutton (108) | 2022 | US | hypothetical population =100,000 | Markov model | Cost-Effectiveness of Hepatitis B Testing and Vaccination of Adults Seeking Care for Sexually Transmitted Infections | HIV and Hepatitis B | One-time HBV pre vaccination testing in addition to HepB vaccination for unvaccinated adults seeking care for STI would save lives and prevent new infections and unnecessary vaccination, and is cost-saving. |  |
| K M Forbes MRCP (75) | 2008 |  | 107 clients attended |  | The objective of this study was to review uptake of sexually transmitted infection (STI) testing in an outreach clinic for those under 25 in an area | STIs | This service has successfully improved access to STI screening |  |
| Thu-Ha Dinh (51) | 2013 | South Africa | 2379 maternity clinical records | a multistage, purposeful sampling strategy | Integration of Preventing Mother-To-Child Transmission of HIV and Syphilis Testing and Treatment | HIV and Syphilis | infection prevalence at delivery was 14% for HIV and 5% for Syphilis.  Integration and provision of a package of HIV and syphilis testing at the first antenatal care (ANC) visit and decentralizing treatments of both infections to primary care settings could increase the coverage of testing and treatment services, thus enhancing the effectiveness of current programs eliminating mother-to-child transmission of HIV and syphilis. |  |
| Luc BÉHANZIN (125) | 2013 | Benin | female sex workers | A cluster sampling  intervention | the prevalence of HIV and sexually transmitted infections among female sex workers | HIV/STI | In Cotonou from 1993 to 2008, there was a significant decrease in HIV, gonorrhea and chlamydia prevalence |  |
| John Bosco Alege (29) | 2025 | Uganda | The study participants (Key Informants)  were both clinical and administrative health workers involved in the delivery of Hepatitis B, C, and HIV services | exploratory qualitative descriptive study | assess barriers and facilitators of integrated viral hepatitis B C and HIV care model to optimize screening uptake among mothers and newborns at health facilities | Hepatitis B C and HIV | High burden of hepatitis B infection, team spirit by the health workers, reduced long waiting time, availability of medical products such as HBV and HCV test kits, integration of HBV and HIV into Health Management Information System (HMIS) 2 data collection tools and availability of support from implementing partners such as Infectious Disease Institute which offered mentorship and training on integration and support supervision.. | Knowledge gaps among healthcare workers, limited  Health education, Language barriers that made communication between health workers and mothers difficult,  constant stock out of test HBV kits, no supplies for HCV kits, and inadequate staffing |
| Y. Lafort (119) | 2002 | Belgium | - | Congress | Investigation of synergistic effects of STI  Control and HIV prevention | HIV, HCV | 1. synergistic impact on HIV prevention  2. improved STI case management and enhanced behavioral change  3. enhance access to hard-to reach groups by the incentive of free care and free condoms | - |
| Annelies Van Den Heuvel (58) | 2019 | multiregional | 400 specimens | diagnostics assessment | the laboratory-based performance of four dual HIV/Syphilis RDTs Laboratories, was evaluated in comparison with a standard reference testing algorithm for HIV and syphilis | HIV/syphilis | 1. increase the accessibility of HIV/Syphilis diagnosis and treatment for difficult to reach populations in the world is promising | 1. need to assess the feasibility and acceptability among health workers |
| P Scott (101) | 1995 | Jamaica |  |  | seeks to promote sexual behavior change, increase condom use, and improve STD diagnosis and treatment | HIV and STI | Despite initial concerns that the integrated program would undermine the quality of family planning services, staff report that their capacity to counsel clients has been enhanced.to change sexual behavior and empowered to refuse unsafe sex. |  |
| Berthollet Bwira Kaboru (127) | 2006 | Zambia | 152 biomedical health practitioners (BHPs) and 144 traditional health practitioners (THPs) | a cross-sectional | explore biomedical and traditional health practitioners' experiences of and attitudes towards collaboration and to identify obstacles and potential opportunities for them to collaborate regarding care for patients with sexually transmitted infections (STIs) and HIV/AIDS. | STIs and HIV/AIDS |  | The study showed a very low level of experience of collaboration. Obstacles  to collaboration were identified at the policy level in terms of legislation and logistics. Lack of trust in THPs by individual BHPs was also found to inhibit collaboration. |
| Jana Jarolimova (60) | 2025 | KwaZulu-Natal, South Africa, Durban | Females | pilot cluster-randomized  controlled trial with randomization at the level of the salon | To address barriers to STI care, we are implementing STI testing  integrated with HIV prevention and contraceptive services | STI& HIV | we reached women with risk factors for STIs and HIV and found a high uptake of STI testing, high rate of treatment completion, and high prevalence of curable STIs |  |
| Mathurin Pierre Kowo (34) | 2025 | Cameroon | people with HIV in 11 HIV clinics | longitudinal demonstration study | Integrating hepatitis C testing and treatment into routine HIV  care | HCV& HIV | Our study demonstrates the feasibility of integrating HCV rapid antibody testing and treatment into routine HIV care by  GPs in Cameroon, yielding new HCV diagnoses and high cure rates |  |
| Yali Luo (86) | 2025 | China | pregnant women | longitudinal study | integrated and standardized services to eliminate mother-to-child transmission of sexually transmitted infections | syphilis and HBV | increased coverage of syphilis treatment for pregnant women living with syphilis, as well as increased coverage of antiviral treatment for high-risk pregnant women living with HBV. services free of charge,  in addition to regular ANC services and continuous follow-up throughout the pregnancy. |  |
| Lucie Sabin (111) | 2025 | Nepal | populations of 752,506 pregnant women | Economic evaluation | Cost-effectiveness of integrated maternal HIV, syphilis, and  hepatitis B screening | HIV& HBV& syphilis | Our modelling analysis showed that dual-integrated screening for HIV and syphilis was highly cost-effective  when compared to current strategy of screening for HIV only (ICERs of US$18). Triple-integrated antenatal screening for HIV, syphilis, and hepatitis B was highly cost-effective compared with dual-integrated strategy with an ICER of US$114. |  |
| Maryam Shahmanesh (82) | 2024 | ural KwaZulu-  Natal, South Africa | 1743 people | randomized factorial trial | integrating HIV prevention within sexual reproductive health services | HIV& STI | STI testing and sexual and reproductive health services create demand for serostatus neutral HIV prevention in adolescents and young adults in Africa. STI testing and integration of HIV and sexual health has increased the potential to reach those at risk. |  |
| Huei-Jiuan Wu (112) | 2024 | Taiwan | MSM people | Economic evaluation | integrating simplified HCV testing into HIV pre-exposure prophylaxis (PrEP) and treatment services | HIV& HCV | Our analysis shows that integrating simplified HCV  testing into HIV prevention and care services would be a  cost-effective strategy for Taiwanese MSM. |  |
| Luh Putu Lila Wulandar (90) | 2024 | Indonesia | 25 key stakeholders | qualitative study | Challenges to integrating programs for the  elimination of mother-to-child transmission of  HIV, syphilis, and hepatitis B into antenatal  care | HIV& HBV& syphilis |  | stock-outs of  rapid test reagents which were particularly most frequent and for longer durations for syphilis  and hepatitis B, high staff turnover, lack of staff training, the complexity  and time needed to record the data on women’s characteristics, risk behaviours, and  discrepancies  in program coverage data from different divisions of the district health office involved in the reporting system, high levels of stigma, challenges in notifying partners, and inadequate reporting and referral of women |
| Thembelihle Zuma (35) | 2024 | rural  KwaZulu‑Natal, South Africa | 1743 men  and women | quantitative and qualitative | implementation  and delivery of HIV prevention services  integrated within sexual reproductive  health | HIV& STI | The intervention was feasible and acceptable to young people and intervention implementing teams. In  particular, the STI testing and SRH components of the intervention were popular | The study found that it  was important to incorporate familial support into interventions for young people's sexual health. Moreover, it was found that psychological and social support was an essential component to combination HIV prevention packages for young people |
| Preeti Manavalan (45) | 2025 | Florida | People refer to 3 STI clinics for HIV care. | Intervention study | Acceptability of Multilevel Sexual Health Interventions and  Sexually Transmitted Infection Screening and Testing  Among Persons with HIV Across Three Clinical | HIV& STI | Acceptability of all interventions was high. The proportion of recommended tests completed was high, although only a subset of at-risk individuals completed rescreening. About 11.9% of rectal samples were positive for chlamydia, and 6.5% of pharyngeal samples were positive for gonorrhea. |  |
| Kristal Scott (83) | 2024– | - | 882 HIV tests- 754 syphilis tests | retrospective review | HIV and syphilis screening, incidence of positive tests, and proportion of patients linked to care. Secondary outcomes included pre-exposure prophylaxis (PrEP) referral and successful linkage rates for HIV-negative syphilis-positive patients | HIV /syphilis | 1.increase screening and early detection for HIV and syphilis.  2.Implemented within the existing emergency department (ED) infrastructure  3.Enables earlier detection and potential for timely intervention | Low referral rate to PrEP and poor follow-up attendance among eligible patients. |
| Lucie SabinI (113) | 2024 | Nepal | 12 in-depth interviews with pregnant women, 10 with their husbands and 4 with mothers-in-law. 7 health workers and 4 decision-makers. | qualitative study | the knowledge, attitudes, and perceptions of pregnant women, their families, healthcare providers and policymakers on integrated prenatal screening. | HIV, syphilis, and hepatitis B | Early Detection and Treatment, Resource Optimization, Cost Efficiency for Patients (in Public Sector), Potential to Normalize STI Testing | Unequal Service Availability, Budget and Financing Constraints, Stigma and Cultural Barriers, Referral System Issues |
| Page, Kathleen R (61) | 2024 | USA (Baltimore) | 720 PWID cohort participants across 12 neighborhoods | Cluster-randomized trial | \| To evaluate  whether an  Integrated Care  Van improves  access to  services and  health outcomes  among PWID \| \| --- \|  \|  \| \| --- \| | HIV, HCV, STIs, opioid use disorder, wound care | - Brought multiple services directly to communities  - Addressed various health needs in one location | - Low engagement from the target cohort (only 7.2% used ICV)  - No significant impact on composite health score |
| Chloe Orkin (84) | 2024 | United Kingdom and the USA | >14 million individuals | Editorial Commentary | To compare the efficacy and cost-effectiveness of combined (triple) testing for HIV, HBV, and HCV versus stand-alone HIV testing, and advocate for integrated Blood-borne virus testing | HIV, HBV, HCV | - Higher case detection (e.g., 5 HBV & 3 HCV per each HIV case)  - Reduces stigma  - Cost-effective  - Increases linkage to care  - Supports health equity  - Enables future integration (e.g., syphilis) | - Operational/logistical challenges (e.g., training staff)  - Triple tests not yet WHO-approved  - Implementation gap despite evidence (slow policy translation) |
| Ruby Massey (126) | 2024 | United States | Patients attending the Sexual Wellness Clinic after emergency departments referral | Retrospective study | To assess patient characteristics and outcomes in an emergency department-linked sexual wellness clinic | STIs (chlamydia, gonorrhea, syphilis, trichomonas), HIV, HCV | - One-stop access to STI testing, treatment, and HIV prevention (PrEP)  - Same-day PrEP initiation  - Linkage to primary care and social services  - Improved coordination via standardized EHR | - - Dependence on emergency departments  Referral pathways may limit reach  - Follow-up data on PrEP continuation lacking |
| Saugat Karki (105) | 2024 | USA | Clinicians + patients | Mixed-methods (pre–post) | Evaluate the feasibility and impact of integrating CDC guidelines for gonorrhea, HIV screening, and PrEP | Gonorrhea, HIV | Streamlined care via electronic health records  Improved adherence to CDC guidelines  Increased HIV screening and PrEP offering  Efficient workflow integration | Limited to one clinic setting |
